# Nanoparticles Engineering by Pulsed Laser Ablation in Liquids: Concepts and Applications

**DOI:** 10.3390/nano10112317

**Published:** 2020-11-23

**Authors:** Enza Fazio, Bilal Gökce, Alessandro De Giacomo, Moreno Meneghetti, Giuseppe Compagnini, Matteo Tommasini, Friedrich Waag, Andrea Lucotti, Chiara Giuseppina Zanchi, Paolo Maria Ossi, Marcella Dell’Aglio, Luisa D’Urso, Marcello Condorelli, Vittorio Scardaci, Francesca Biscaglia, Lucio Litti, Marina Gobbo, Giovanni Gallo, Marco Santoro, Sebastiano Trusso, Fortunato Neri

**Affiliations:** 1 Department of Mathematical and Computational Sciences, Physics and Earth Physics, University of Messina, Viale F. Stagno D’Alcontres 31, I-98166 Messina, Italy; ggallo@unime.it (G.G.); fneri@unime.it (F.N.); 2 Department of Technical Chemistry I and Center for Nanointegration Duisburg-Essen, University of Duisburg-Essen, Universitätsstrasse 7, 45141 Essen, Germany; bilal.goekce@uni-due.de (B.G.); friedrich.waag@uni-due.de (F.W.); 3 Department of Chemistry, University of Bari, Via Orabona 4, 70126 Bari, Italy; alessandro.degiacomo@uniba.it; 4 CNR-NANOTEC, c/o Department of Chemistry, University of Bari, Via Orabona 4, 70126 Bari, Italy; marcella.dellaglio@imip.cnr.it; 5 Department of Chemical Sciences, University of Padova, Via Marzolo 1, 35131 Padova, Italy; moreno.meneghetti@unipd.it (M.M.); francesca.biscaglia@unipd.it (F.B.); lucio.litti@unipd.it (L.L.); marina.gobbo@unipd.it (M.G.); 6 Department of Chemical Sciences, University of Catania, V.le A. Doria 6, 95125 Catania, Italy; gcompagnini@unict.it (G.C.); ldurso@unict.it (L.D.); marcello.condorelli@unict.it (M.C.); vittorio.scardaci@unict.it (V.S.); 7 Department of Chemistry, Materials, Chemical Engineering, Politecnico di Milano, Piazza Leonardo da Vinci 32, I-20133 Milano, Italy; matteo.tommasini@polimi.it (M.T.); andrea.lucotti@polimi.it (A.L.); chiara.zanchi@tiscali.it (C.G.Z.); 8 Department of Energy & Center for NanoEngineered Materials and Surfaces—NEMAS, Politecnico di Milano, Piazza Leonardo da Vinci 32, I-20133 Milano, Italy; paolo.ossi@polimi.it; 9 STMicroelectronics S.R.L., Stradale Primosole 37, 95121 Catania, Italy; marco.santoro@st.com; 10 CNR-IPCF Istituto per i Processi Chimico-Fisici, 98053 Messina, Italy; trusso@ipcf.cnr.it

**Keywords:** colloids, laser synthesis, plasmonics, sensing, biomedicine, catalysis, pollutants

## Abstract

Laser synthesis emerges as a suitable technique to produce ligand-free nanoparticles, alloys and functionalized nanomaterials for catalysis, imaging, biomedicine, energy and environmental applications. In the last decade, laser ablation and nanoparticle generation in liquids has proven to be a unique and efficient technique to generate, excite, fragment and conjugate a large variety of nanostructures in a scalable and clean way. In this work, we give an overview on the fundamentals of pulsed laser synthesis of nanocolloids and new information about its scalability towards selected applications. Biomedicine, catalysis and sensing are the application areas mainly discussed in this review, highlighting advantages of laser-synthesized nanoparticles for these types of applications and, once partially resolved, the limitations to the technique for large-scale applications.

## 1. Introduction

The intrinsic properties of nanoparticles (NPs), possibly combined with other materials, disclose many applications where one can achieve miniaturization (e.g., of electronic equipment), weight reduction (as a result of an increased material efficiency) and/or improved functionalities of materials (e.g., higher durability, conductivity, thermal stability, solubility, reduced friction, selective molecular detection) [1,2]. The remarkable size-tunable properties of nanomaterials produced by laser–matter interaction (e.g., size distribution, agglomeration state/dispersion, crystal structure, surface area and porosity, surface charge, shape/morphology, dissolution/solubility) make them a hot research topic in material science, with far-reaching applications, ranging from quantum computers to cures for cancer [3,4].

Bottom-up and top-down procedures are the two approaches used for the synthesis of nanomaterials [5]. The first include the miniaturization of materials and components (up to the atomic level) with subsequent self-assembling that leads to the formation of nanostructures. Typical examples are the formation of quantum dots during epitaxial growth, or the production of NPs as colloidal dispersions by chemical approaches [6]. Instead, top-down approaches use macroscopic starting structures that are externally controlled by the processing down to nanostructures. Typical examples are etching (controlled by masks), ball milling, metal-organic vapor phase epitaxy and the application of severe plastic deformations [7].

There are many literature papers on laser interaction with hard, soft and smart materials, targeting future applications in the fields of energy production (nano-energy) and biomedicine [8], as well as recent progress in the understanding of the fundamental mechanisms involved in laser processing [9]. Such laser techniques are interesting in many regards, as they enable the processing of photovoltaic cells [10], thermoelectric materials and devices [11], micro and nanosystems for energy storage and conversion [12,13], biodegradable and biocompatible NPs for food packaging [14] as well as vectors for drug and gene delivery [15,16]. The interest towards pure and electrostatically stabilized nanocolloids is well recognized [17,18,19,20,21]. Although nanotechnologies and nanomaterials have much potential to introduce innovative products and production processes in the food industry and in the biomedical field, they are facing major challenges in being cost-effective in the production of e.g., edible and nontoxic nano-delivery systems, or effective formulations that are safe for human consumption and drug treatments [22].

Laser synthesis of colloids, powered by robust, high-power lasers, appears to be a key enabling process that is chemically clean and environmentally friendly, and appealing [23,24] for industrial manufacturing of functional nanomaterials while being useful in many different areas, such as: hydrogen generation [16], hydrogen storage [25], heterogeneous catalysis using colloidal high-entropy alloy NPs [26,27], anticancer [28] and antimicrobial [29] research, drug monitoring [30], additive manufacturing applications [31,32], and nonlinear nanophotonics [33]. In addition, NPs prepared by the Laser Ablation in Liquids (LAL) have been recently used for various and unique applications like friction reduction [34], solar nanofluids [35,36], optical limiting devices [37,38] and so on.

The generation of NPs with LAL still has some challenging aspects, such as the fabrication of NPs with a specific size and shape, the reduction of polydispersity and the increase of productivity [39]. Despite some unsolved problems of a physical, chemical and technical nature, several strategies have been proposed to overcome the above issues, including the selection of the appropriate liquid or stabilizing agent, the optimization of the focusing conditions and liquid levels, as well as the adoption of scanning and fluid dynamics strategies by different liquid handling configurations [40,41,42,43,44] and the postirradiation of colloids [45].

To explore the wide range of opportunities that LAL brings, the unique properties of femtosecond lasers is a further hot research topic, not only to increase NP production, but also to generate structural modifications and new material phases [46,47]. As reported by Streubel et al. [48], low productivity shortcomings in LAL have been almost entirely remedied, reaching NP production rates of several grams per hour. In addition to LAL, Laser Melting in Liquids (LML), Laser Fragmentation in Liquids (LFL) as well as pulsed Laser Photoreduction/oxidation in Liquids (LPL), offer alternative routes to obtain colloids with controlled NP sizes. Nevertheless, an industrial series product, or manufacturing process based on laser-synthesized particles is yet to come. Together with the development of new strategies to increase NP productivity, future efforts should be directed to further improve the surface properties of the produced NPs, since each application requires a different surface/interface chemistry. This would ensure reaching an advanced stage in those applications where the surface processes determine the performance of the final devices [49].

**Figure 1:**
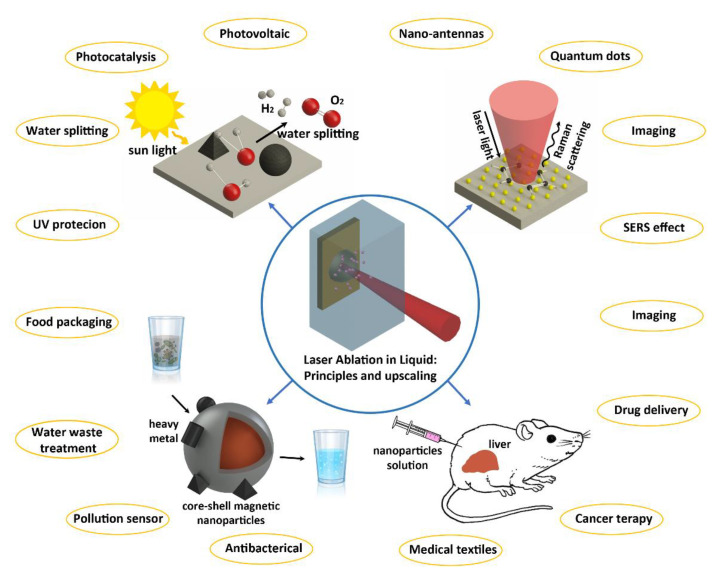
Schematic showing the outline of the review. From the fundamentals and up-scaling, in the center, arrows point outwards to the four application areas.

In this review, we first give an overview of the fundamentals of pulsed laser ablation synthesis. Moreover, we aim to display a combined picture of the several processes implied by LAL [50,51], remarking, with respect to previous reviews [23,39,52,53,54], the high complexity and multiple time-scale transient physics and chemistry typical of LAL. We point at this target by taking into account different experimental conditions. The fundamental mechanisms of LAL are analyzed in depth, to stimulate concepts that are suitable to develop high performance devices based on the peculiar properties that the nanomaterials produced by LAL can offer. In the second part of this review, we outline selected applications of LAL in biomedicine, catalysis and sensing. We outline the advantages of laser synthesized NPs for these applications (see Figure 1) and the issues that have to be solved to approach the high degree of process control that is required in industrial applications.

Throughout the review we pointed at two main objectives. First, to offer an analytical overview that is updated and complete on the principles of LAL method, as well as on the role that is played on the synthesis of NPs by process parameters, both laser and geometry related, and by the effects specifically depending on the liquid. Second, we describe the applications we presently believe are most promising in the field: these include plasmonic-related sensing and nanomedicine, and catalysis.

## 2. LAL Synthesis Methods: Principle, Process Parameters and Liquids Effects on Nanoparticle Formation

### 2.1. Mechanism in ns-Pulsed Laser Ablation in Liquid for the Production of Metallic Nanoparticles

Laser Ablation in Liquid (LAL) for the production of nanostructures is based on the ejection of material by a laser pulse irradiating a solid target immersed in liquid. The laser matter interaction and the consequent ablation are strongly dependent on the irradiance and the duration of the pulse, on the background liquid, on the sample geometry and morphology as well as on the focusing condition. A full description of the LAL, at different experimental conditions, is beyond the scope of this paper and the interested reader can refer to the following review papers: [39,52,55], and references therein. Here, in order to offer a general viewpoint on the complexity of the sequence of the processes involved in LAL, ns-LAL, for the production of metal NPs in water, will be discussed.

**Figure 2:**
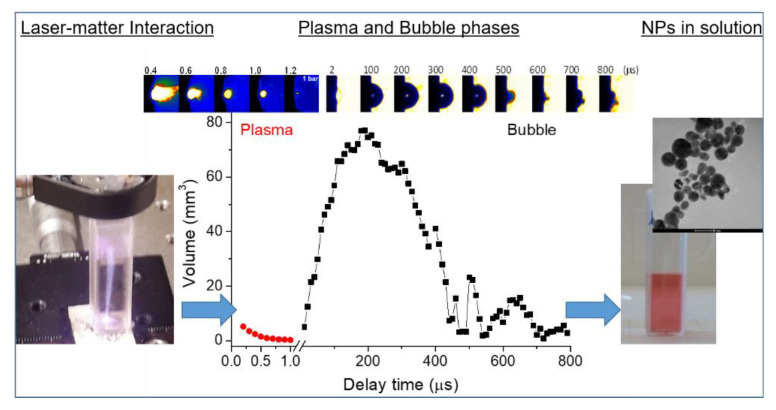
Different steps of Laser Ablation in Liquid (LAL). The picture of laser-matter interaction refers to a ns-laser focused on Pt target in water. The plasma and bubble images and volumes as functions of the delay time since the laser pulse have been acquired with optical emission imaging and shadowgraphy, respectively (Al target in MilliQ water, *E*_laser_ = 270 mJ, laser crater size = 1.6 ± 0.2 mm, *λ*_laser_ = 532 nm, laser pulse duration = 6 ns, laser frequency = 10 Hz). The nanoparticles (NPs) visible in cuvette and micrograph image are Au NPs.

As it has been clearly established, ns-PLAL is based on a sequence of different processes: laser ablation and plasma induction, energy exchange from plasma to the liquid and consequent generation of the cavitation bubble and release of particles from the bubble to the solution. In Figure 2 the different LAL steps are summarized.

Laser-matter interaction: The basic mechanisms inducing the laser ablation in liquid, as well as their dependence on laser pulse properties, do not differ notably, with respect of laser ablation in gaseous environment [54]. In the case of ns-laser ablation, just a portion of the laser pulse reaches directly the target surface, while most of the laser pulse is spent in electron heating by inverse bremsstrahlung. This implies that the ablated material is converted to a plasma phase during the laser pulse irradiation. Differently to what can be observed in a gas background environment, as a consequence of the water incompressibility, the ablated material is strongly confined and in turn reaches high density. This effect decreases the penetration of the laser through the plasma during the initial stage of expansion, inducing the propagation of gradient of temperature in the ejected material, i.e the plasma. For what concerns the type of target used, it should be considered that morphological, electrical and optical properties of the target affect the initial interaction of the material with the laser pulse. For example, a metallic target allows the use of lower ablation irradiance with respect to the semiconductor, if we neglect the difference in reflectivity. In any case if an irradiance well above the breakdown threshold is used, the ablation can be assumed congruent, and similar plasma behavior can be observed.

The mechanisms concerning the evolution of the ejected material after laser ablation are strongly correlated to the duration of the laser pulse. Ideally, with a laser pulse with a shorter duration than a few tens of fs, the effect of the interaction of the laser pulse with the ejected material can be neglected and the evolution of the ablated material can be investigated with atomistic models [56]. On the contrary, when the laser pulse is long enough (as in the case of ns) to effectively interact with the ablated material, inducing further ionization and electron heating, the role of the plasma becomes dominant and kinetics models coupled with fluidynamics are required for a full description of the phenomenon [57]. In this review, when describing fundamental aspects, we will refer to ns-ablation, while a brief discussion on ultrashort ablation is reported in Section 2.2.

Plasma phase: The plasma phase plays an important role during ns-LAL because the plasma is the source of material that allows the formation of NPs. Plasma phase during ns-LAL can be investigated with Optical Emission Spectroscopy (OES) in order to estimate the plasma parameters such as electron number density, *N_e_*, atomic number density, *N_a_*, and excitation temperature, *T*, as well as the features of plasma dynamics after laser ablation. The emission spectrum of the Laser Induced Plasma (LIP) under water is characterized mainly by continuum radiation with possibly some resonance peaks of the element constituting the target [40]. The absence of the typical atomic spectral lines of the LIP expanding in a gaseous environment is due to the high density reached by the ablated matter under liquid confinement as discussed in [40,41,42]. In this case, the emission spectrum has a Planck-like shape that has been ascribed to the radiative recombination and that in turn is proportional to the blackbody law, although the spectrum is not related to any blackbody system. To better understand this crucial point, let us consider blackbody (BB) power density in the Wiens’ approximation at *hν*/*kT* >> 1, which is valid for the range 400–500 nm and *T* ~ 6000 K:

(1) $$B_{v}(v,T)=\frac{2hv^{3}}{c^{2}}\frac{1}{\exp\left(\frac{hv}{kT}\right)-1}\approx \frac{2hv^{3}}{c^{2}}\exp\left(-\frac{hv}{kT}\right)\propto v^{3}\exp\left(-\frac{hv}{kT}\right)$$

where *h* is the Planck constant, *ν* is the frequency, *c* is the light speed, *k* is the Boltzmann constant and *T* is the temperature.

On the other hand, the radiative recombination power density is given by the following equation [6]:

(2) $$\rho_{{}^{p}}^{R}(v,T)=2\sqrt{\frac{2}{\pi }}\frac{h^{4}}{m_{e}^{3/2}c^{2}}2p^{2}{\left(\frac{1}{kT_{}}\right)}^{3/2}\exp\left(-\frac{hv-(E_{i}-E_{p})}{kT}\right)\sigma_{ion}(p,v)v^{3}N_{e}^{2}$$

where *m_e_* is the electron mass, *E_i_* and *E_p_* are the ionization energy and the energy of the atom in the level p, respectively, *N_e_* is the electron number density and *σ*_ion_ (*p*, *ν*) is the photoionization cross section.

Equation (2) can be arranged as:

(3) $$\rho_{{}^{p}}^{R}(v,T)=2\sqrt{\frac{2}{\pi }}\frac{h^{3}}{m_{e}^{3/2}}2p^{2}{\left(\frac{1}{kT_{}}\right)}^{3/2}\sigma_{ion}(p,v)N_{e}^{2}\frac{hv^{3}}{c^{2}}\exp\left(-\frac{hv-(E_{i}-E_{p})}{kT}\right)$$

Considering the high density due to the plasma confinement in water, only a few levels are available and they are very close to the ionization energy [43]:

(4) $$\exp\left(-\frac{hv-(E_{i}-E_{p})}{kT}\right)\approx \exp\left(-\frac{hv}{kT}\right)$$

Consequently, Equation (3) becomes:

(5) $$\begin{array}{l} \rho_{{}^{p}}^{R}(v,T)=2\sqrt{\frac{2}{\pi }}\frac{h^{3}}{m_{e}^{3/2}}2p^{2}{\left(\frac{1}{kT_{}}\right)}^{3/2}\sigma_{ion}(p,v)N_{e}^{2}\frac{hv^{3}}{c^{2}}\exp\left(-\frac{hv}{kT}\right)=\\ =\sqrt{\frac{2}{\pi }}\frac{h^{3}}{m_{e}^{3/2}}2p^{2}{\left(\frac{1}{kT}\right)}^{3/2}\sigma_{ion}(p,v)N_{e}^{2}B_{v}(v,T) \end{array}$$

The photoionization cross section at high density conditions, shows a smooth dependence on wavelength [42], so that its contribution can be considered constant in the range of photon energy investigated in the visible range, i.e., 1–3 eV. In the frame of this assumption and since, at fixed time *N_e_* and *T* are constant, Equation (5) becomes:

(6) $$\rho_{{}^{p}}^{R}(v,T)=G(T,N_{e})\cdot B_{v}(v,T)$$

where *G* is a constant with respect to wavelength at each time of plasma lifetime since it represents a function depending on detection efficiency, on the electron number density and on plasma temperature, but not on the wavelength. Therefore, within the previous approximation, by integrating Equation (6) with d*ν*, a Planck-like distribution can be obtained. This may justify the observation of the Planck-like distribution of plasma emission in several experiments [58,59,60] although the plasma is not a blackbody.

Recently temporally and spatially resolved OES has been applied during LAL for the production of metallic NPs in order to estimate the temperature and density maps with data processing as described in detail in [60].

**Figure 3:**
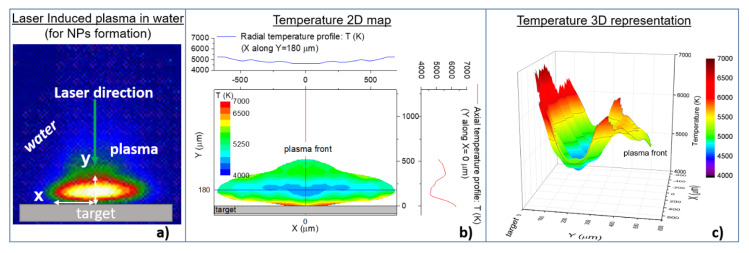
(**a**) Image of laser-induced plasma on Al target immersed in water, acquired with ICCD camera. (**b**) Plasma temperature 2D map calculated as reported in [43] (Al target in MilliQ water, *E*_laser_ = 270 mJ, laser crater size = 1 ± 0.2 mm, *λ*_laser_ = 532 nm, laser pulse duration = 6 ns, laser frequency = 10 Hz, delay time = 40 ns, gate width = 20 ns). (**c**) 3D representation of plasma temperature in (**b**).

As an example, Figure 3 shows the temperature map of a plasma induced on an aluminum target immersed in water. By the inspection of the figure it can be observed that in the core of the plasma, where the number density is very high [60,61], there is a decrease in temperature beyond the condensation temperature of most of the metals. As a matter of fact, the high pressure of the plasma at this condition, i.e., under water confinement, allows for condensation at higher temperatures than those in standard condition. This observation indicates that NPs can be formed in the bulk of the plasma and not only in the border of the plasma where the plasma cools down fast as a consequence of the rapid exchange of energy between the plasma and the surrounding liquid. It may be assumed that also the material on the plasma border condensates in particle, but being this zone of the plasma out of equilibrium, it produces particles with various sizes and shapes. On the contrary, particles formed in the bulk of the plasma, being the result of thermodynamic equilibrium between the processes of growth and the evaporation, are characterized by a spherical shape with narrow size distribution.

The mechanism of growth in the plasma phase during ns-LAL has been investigated with a theoretical model in [61] where the competition between thermodynamic condensation and electrostatic growth has been investigated. The result shows that at the electron number density usually found in ns-LIP in water, the charging of seeds and particles occurs at ps-time scale. This observation suggests that as soon as small clusters are formed because of the high-density gradient at the initial stage of laser ablation, electrons attach. Consequently, the particles become negatively charged and attract ions in the plasma. Electron charging and ion implantation allows the particles to grow until the rate of growth is balanced by the evaporation process due to the high temperature in the plasma (4000–6000 K). In this scenario, the equilibrium between electrostatic growth and thermodynamic evaporation set the NPs dimensions and induces the typical spherical shape observed after the LAL process.

Recently it has been shown, that the charging effect of the plasma is involved also in the subsequent steps of LAL process [62,63]. As a matter of fact, when the particle exits the plasma, it is still with an excess of electrons. This charge, in the order of a few nC for the entire NPs production per laser shot, allows the repulsion between the particles and preserves the NPs from massive aggregation.

**Figure 4:**
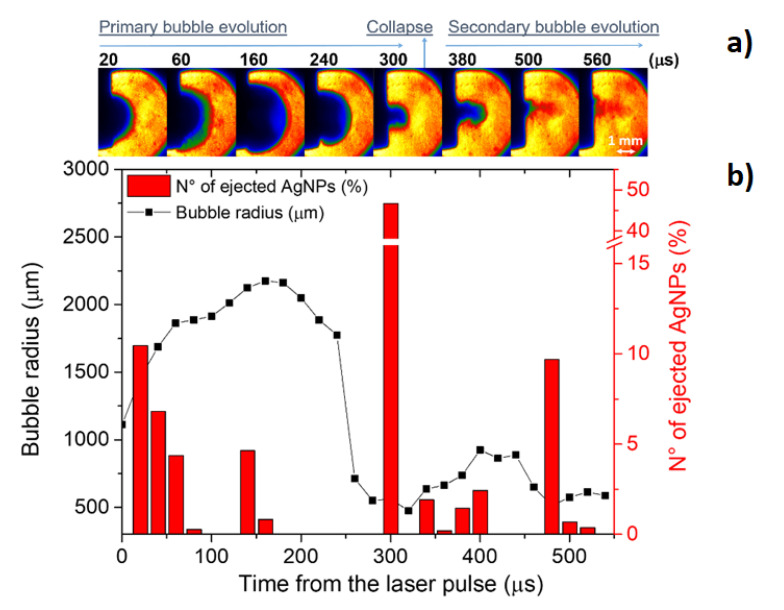
Time resolved shadowgraphy images of the laser induced bubble on Ag target immersed in water (**a**) and the corresponding bubble radius compared with the number of ejected Ag NPs during the bubble evolution (as calculated in [44]) (**b**). Experimental conditions: laser energy = 108 mJ, laser frequency = 2 Hz, laser crater size = 1 ± 0.2 mm, ICCD gate width = 20 µs and 5 accumulations for each image.

The cavitation bubble: The fast transfer of energy from the plasma to the surrounding water induces the formation of a thin layer of vapor around the plasma border with high temperature and high pressure. In order to reach the equilibrium with the liquid, the vapor expands, producing a cavitation bubble. In the upper part of Figure 4, an example of the temporal evolution of a laser-induced bubble during silver ablation is reported. The bubble expands until it reaches the equilibrium with the surrounding liquid. Due to the fast expansion, the liquid at the border of the cavitation is compressed and when the pressure of the bubble reaches the minimum at the maximum of the expansion, the bubble is compressed and its shrinking stage begins. During this stage, the bubble increases its pressure again and impacts the target, eventually pushing back the material to the target surface. There are several models describing the evolution of a laser-induced bubble [64,65]. Most of these descriptions are based on a theoretical model simulating the bubble radius evolution, assuming the mass and momentum conservation equations in the liquid phase, and that the vapor pressure inside the bubble is balanced by the pressure on the liquid side of bubble wall [66]. As an example, neglecting the evaporation/condensation effects as the time associated to bubble dynamics is shorter than that of these processes, the Keller–Miksis formulation can be applied with the following equations [66]:

(7) $$\left(1-M\right)\rho_{l}R\ddot{R}+\frac{3}{2}\left(1-\frac{M}{3}\right)\rho_{l}{\dot{R}}^{2}=\left(1+M+\frac{R}{c_{l}}\frac{d}{dt}\right)\left(p_{g}\left(t\right)-\frac{2\sigma_{l}}{R}-4\eta_{l}\frac{\dot{R}}{R}-p_{\infty }\right)$$

where *R* is the radius of the bubble and each dot indicates a time derivative, *ρ_l_* is the liquid water density, *M* is the bubble-wall Mach number, *c_l_* is the sound speed in the liquid, *p_g_* and *p_∞_* are, respectively, the gas pressure inside the bubble and the background static pressure and *σ_l_* and *η_l_* are, respectively, the surface tension and dynamic viscosity of liquid water. The equation can be resolved considering the weakly compressible equation of state for the liquid density, and assuming an adiabatic van der Waals equation of state for a spherical bubble so that the vapor pressure and temperature are related to the radius by the following equations:

(8) $$p_{g}\left(t\right)=\left(p_{\infty }+\frac{2\sigma_{l}}{R}\right){\left(\frac{R_{\infty }^{3}-h^{3}}{R^{3}-h^{3}}\right)}^{\gamma }$$

(9) $$T\left(t\right)=T_{\infty }{\left(\frac{R_{\infty }^{3}-h^{3}}{R^{3}-h^{3}}\right)}^{\gamma -1}$$

where *R*_∞_ is the radius at which the pressure inside the bubble corresponds to *p*_∞_, *h* is the radius determined by the excluded volume of the water molecules and *γ* = *C_p_*/*C_v_* = (2 + *N*)/*N* is the ratio of the specific heats, with *N* the number of degrees of freedom. The model starts assuming the initial radius equal to that of the laser spot and the gas inside the bubble in thermal equilibrium with the liquid. Although this simulation does not take into account the interaction of laser-induced plasma with the vapor inside the bubble, it provides an acceptable qualitative description of bubble evolution.

As a result of the charging effect on the NPs produced in the plasma phase due to the electrostatic repulsion, NP clouds expand as well. If the NPs store enough charge immediately after the plasma stage, the induced electrostatic pressure can be higher than the pressure at the water/vapor boundary and NPs exit from the cavitation bubble and are released in solution. Recently, this phenomenon has been investigated in detail in [62] and it has been found that the two main streams of NPs ejection due to the electrostatic repulsion within the particle cloud in the bubble occurs immediately after the beginning of the expansion phase and, in a greater extent, between the collapsing stage and the bubble rebound [62]. Figure 4 shows the number of ejected AgNPs during the cavitation bubble evolution compared with the measured bubble radius, using Equations (8) and (9). The number of the ejected AgNPs has been calculated as reported in [62].

It would be important to underline that the spherical NPs with a size around 10–20 nm are not the only particles produced. Indeed, in terms of mass, the fraction of material converted in this kind of NPs is a minor component. Larger particles are produced at the border of the plasma during the energy exchange from plasma to the surrounding liquid as mentioned below. Other bigger NPs can be the result of aggregation of the spherical NPs during the collapse stage of the bubble. The NPs that are not ejected because of the electrostatic repulsion can remain trapped in the bubble, therefore they can be then compressed, because of the high pressure of the collapse stage, rearranging their structure in aggregates composed by individual spherical NPs. Finally, as an intrinsic result of the laser ablation, debris of several hundred of nm with the same morphology of the irradiated target can be found on the bottom of the ablation cell.

All the discussion here refers to ns laser ablation and it gives a simplified scenario of the processes involved in the production of NPs of high quality in terms of size distribution and chemical purity of the colloidal solution. In order to increase the production rate, in recent years, different approaches have been developed based on K-Hz laser source, ultrashort laser pulse and target shape, as will be discussed in Section 2.2.

### 2.2. Upscaling of Laser Synthesis of Colloids

As can be derived from the previous section, understanding the mechanisms of a process enables its control, which can then lead to a precise experimental design. This section reviews process parameters of LAL that are of great importance for the scale-up of this process [66]. Additionally, cross-dependencies between the single parameters are pointed out.

#### 2.2.1. Scaling and Control Factors for Laser Ablation in Liquids

LAL process parameters are initial, adjustable, and related to components of the process, which are the laser, the components of the optical setup, the ablation chamber, the fluid flow elements, the ablation target, and the liquid environment. The process result of interest is the NP productivity, which can be defined as volume of product achieved in a defined irradiation time (considering energy efficiency, additional reference values like the laser power become relevant as well). In literature, the ablation rate is frequently used for productivity comparisons. It corresponds to the loss of target volume achieved in a defined irradiation time. By assuming a 100% conversion of ablated matter to NPs, the definitions of productivity and ablation rate are identical.

**Figure 5:**
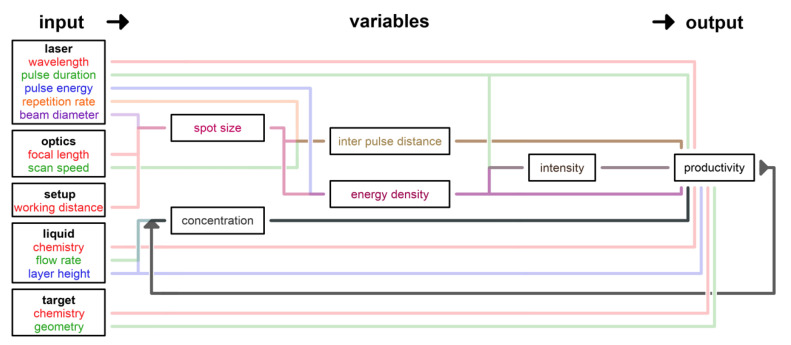
Dependence graph of process parameters, arranged in component clusters, relevant to LAL up-scaling. If not marked differently by an arrow, the flow goes from the left to the right in horizontal direction and from the outside to the inside in vertical direction. Colors were used only for an improved overview. Reprinted with permission from [66].

A process starts with the input of initial parameters and creates an output, which comprises the results. In between those process parameters and results, variables can be defined. They generally depend on multiple process parameters of different components of the setup and bundle their impact on the process. The dependency graph shown in Figure 5 displays the compilation of the process parameters of LAL clustered and assigned to the different components of the LAL setup. In addition, the cross-dependencies of parameters, found in the process variables, and finally the process result(s) are also illustrated in the figure.

The use of different colors supports the readability of the dependency graph. Each time two or more parameters or variables interdepend, their colors add up. For example, the input parameter pulse duration of the cluster laser influences directly the output productivity but also indirectly the variable intensity. The colors of pulse duration (green) and energy density (wine) add up to dark brown, which represents the variable intensity. In the following sections, the impact of single input parameters and also the assembled variables on the NP productivity are evaluated. Note that product properties such as the particle size distribution also represent an output of the process but are not considered in detail here.

Laser Wavelength: The laser wavelength essentially influences the ablation rate in LAL [54,67,68,69,70]. All studies agree independently from the ablated matter, the chosen liquid and the used laser pulse duration on a higher productivity by using IR laser light compared to UV or Vis. The laser fluence was kept at identical or at least comparable values in all studies. However, LAL was applied as a batch approach in all investigations, and NP-induced shielding due to Rayleigh scattering [71] most likely caused the differences in productivity. In addition, a higher absorption cross-section is given for NPs of nearly all metals at UV or Vis wavelengths compared to IR wavelengths [72]. This is strongly indicated by the observed smaller NP diameters at shorter applied wavelengths [54,69,70], which may be the result of fragmentation induced by subsequent laser pulses during batch-LAL.

Schwenke et al. [54] determined the product concentration during picosecond-pulsed LAL at different process times for IR and Vis wavelengths and three different metals. They found that the concentration was comparable for both wavelengths at short process times. However, it increased differently with time or the NP concentration, and less strongly at short wavelengths in all cases. These results clearly demonstrate the shielding effect of the colloidal NPs.

Semerok et al. [73] found higher ablation rates by using shorter wavelengths in the picosecond and nanosecond-pulsed laser ablation in air for several metals. The ablation rate increased stronger for most metals when switching from the Vis to the UV range then from the IR to the Vis range, which was in good agreement with the interband absorption of the targets. Similar results were also found by Stafe et al. [74]. Naturally, the individual light absorption properties of bulk materials should influence the number of absorbed photons of different wavelengths and provide optimums for high ablation rates. However, the optical properties of an irradiated surface significantly depend on its temperature [75], roughness [76,77] and oxidation state/degree [78]. For example, Letzel et al. [79] recently observed a significant increase in the productivity of the laser ablation of silver in water during the first 250 laser pulses irradiating an initially smooth target surface. Temperature, roughness and oxidation state/degree of the target surface change differently strong during LAL and depend strongly on the ablated material. This limits the a priori choice of the most productive wavelength for LAL according to the standard light absorption spectrum of the material to be ablated.

**Figure 6:**
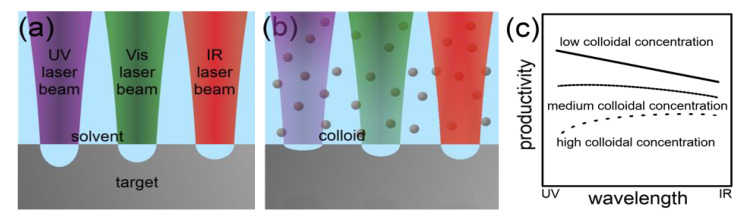
Schematic of the laser ablation of metals in liquids at different wavelengths in absence (**a**) and presence (**b**) of colloidal particles. Equal energy densities were assumed for all laser spots. The diagram in (**c**) (linear scales for both axes) demonstrates the effect of an increasing colloidal concentration on the NP productivity in LAL at different wavelengths. The trends shown in the diagram represent the interpretation of the combined results of the discussed literature in Section 2.2.1. Reprinted with permission from [66].

It may be concluded that higher ablation rates for metals generally occur at UV laser wavelengths due to the interband absorption. However, the productivity at Vis and IR wavelengths can be comparable. A strong dependence on the metal, the laser fluence and also the laser pulse duration exists. In case of high colloidal concentrations, the higher absorbance of short wavelengths by the particles can make LAL of metals at short wavelengths less productive. These relations are summarized in Figure 6. Note that for semiconducting and dielectric materials the initial ablation rate in absence of a light-extinction colloid can be higher at red and near IR wavelengths under specific conditions due to differences in the ablation mechanism compared to metals [80,81].

Laser Fluence: The laser fluence or energy density describes the pulse energy penetrating the area of the effective laser spot on the surface of the ablation target. A variation of the laser fluence may be realized by either varying the pulse energy [67,70,71,82,83,84,85,86,87,88,89,90] or the spot area via the working distance [67,85,91]. Different results by both procedures can occur due to variations of the beam propagation within the liquid phase. Furthermore, the distribution of the laser energy density in the spot of a Gaussian beam differs. Naturally, the ablation rate in LAL follows a logarithmic growth when increasing the laser fluence of femtosecond [90], picosecond [82,88] and nanosecond [85] pulses. At very low values of the laser fluence, a less productive ablation regime prevails [82], which is not considered here. The logarithmic dependence is also known for laser ablation in a gaseous environment [91,92,93,94,95].

The onset of the saturation occurs at lower laser fluences for shorter laser pulse durations [90], which may be due to higher laser intensities and the related optical breakdown of the liquid. The lowered thermal impact on the material, and therefore stronger independence of laser penetration depths and fluence at shorter laser pulse length, may also play a role here. In addition, the type of the ablated material influences the onset of the saturation in the ablation rate [83,85,92] by different laser fluence thresholds and penetration depths. For example, Hahn et al. [83] could not increase the productivity of the laser ablation of silver in water in the pulse energy range of 100 to 500 µJ but nearly tripled the productivity of the ablation of cobalt by increasing the pulse energy from 100 to 200 µJ before reaching saturation. The working distance was kept constant by the authors.

The conventional definition of productivity in LAL (volume ablation per time) used in this manuscript can also be related to the applied laser energy. This extended definition allows an assessment of the energy efficiency, which seems reasonable from an economic and ecological point of view. Neuenschwander et al. [96] demonstrated theoretically and experimentally that a maximum energy efficiency of ultrashort-pulsed laser ablation is reached at an optimum fluence, which is the material-specific threshold fluence of laser ablation multiplied by e^2^. While the authors of the previously mentioned study focused on laser ablation in air, Streubel et al. [44] also verified the correlation for laser ablation in liquids.

**Figure 7:**
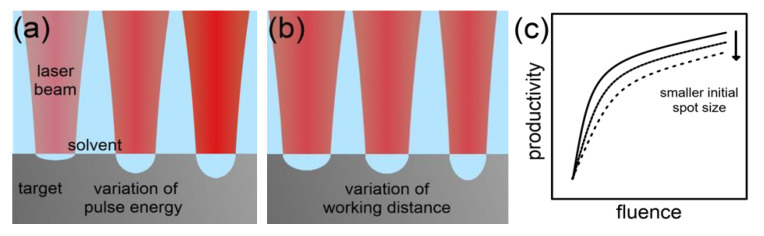
Illustration of the laser ablation of metals in liquids at different fluences varied by the pulse energy (**a**) and the working distance (**b**). The diagram in (**c**) demonstrates how the fluence variation caused by different initial spot sizes affects the NP productivity in LAL in the high-fluence regime. A Gaussian beam profile was assumed. In (**c**), both axes scale linearly. The trends shown in the diagram represent the interpretation of the combined results of the discussed literature in Section 2.2.1. The arrow and the less dense (dashed) line style in (**c**) indicate the trend in productivity induced by a smaller initial spot size. Reprinted with permission from [66].

In conclusion, the volume ablation rate logarithmically scales with the laser fluence. The progress of the logarithmic function further depends on the ablation mechanism, the surrounding liquid, and the target material. If productivity in LAL is further related to the invested laser pulse energy, an optimum fluence can be found. Figure 7 illustrates the typical dependency of the NP productivity on the laser fluence.

Laser Pulse Duration: The mechanism of laser ablation crucially depends on the pulse duration. The influence of moderate fluences on the ablation rate of metals in gaseous atmosphere can be well described by the two-temperature model [97] for femtosecond and picosecond pulses and by a simple evaporation model at longer durations [95]. Extensions and refinements of the two-temperature model even allow accurate predictions of the ablation rate in the femtosecond-pulsed laser ablation of dielectrics [98] or of the low-fluence regime in the picosecond-pulsed laser ablation of metals [99]. Exchanging the gaseous ambient by a liquid causes a more intense cooling of the ablation target and of the laser-induced plasma, which may affect the ablation process but should neither change the principal ablation mechanism nor invalidate the describing models.

Conclusively, a strong impact of the pulse duration on the ablation rate exists. A fair experimental comparison of the ablation rate with laser pulses with durations ranging from some 10 femtosecond to some 100 ns is not accessible due to the lack of suitable laser technology. However, if a wide-range variation of the pulse duration would be possible (keeping the pulse energy constant), a more effective use of the energy of single laser pulses should occur at shorter pulse durations due to the reduced heat loss. However, the optical breakdown of the liquid in front of the ablation target as well as non-linear optical effects such as the optical Kerr leading to filamentation limits the applicable fluence stronger at shorter laser pulses due to their higher intensities.

Riabinina et al. [90] investigated the NP productivity of the LAL of gold in an aqueous sodium citrate solution at different pulse durations between 40 fs and 200 ps but a constant fluence of 50 mJ/cm^2^. An optimal productivity was found at a pulse duration of 2 ps. The authors expected a decrease in the productivity at lower pulse durations to be caused by the optical breakdown of the liquid. A loss of productivity at higher pulse durations was expected to originate from heat losses in the target and laser-induced shielding effects. During their experiments, the laser pulse repetition rate was kept constant and the scanning speed was kept at zero, which makes shielding effects at longer pulse durations realistic. Interestingly, Sakka et al. [100] also observed a decreasing ablation rate at an increasing pulse duration during the nanosecond-pulsed laser ablation of copper in water, although this effect did not occur during laser ablation in air. This finding confirms the origin of a negative impact of an increasing pulse duration on the ablation rate in the presence of the liquid environment. In another study, Dittrich et al. compared the absolute and power-specific productivity of LAL of gold in water for a compact, a middle class and a high-end laser system [101]. The authors applied LAL in a liquid flow and with sufficient laser scanning to limit shielding effects. They found that even though the absolute productivity was the lowest for the low-power, compact laser system, power-specific productivity was much higher compared to the other systems. All three laser systems differed in the laser pulse duration, which was 1 ns for the compact, 5 ns for the middle-class and 3 ps for the high-end laser system, respectively.

Further details on the effect of the pulse duration on the ablation rate can be derived from studies on the laser ablation of metals in air and vacuum. Schille et al. [102] found a slight decrease in the ablation rate of copper during an increase of the pulse duration from 200 fs to 4 ps. A further increase of the pulse duration up to 10 ps significantly reduced the ablation rate. As demonstrated by Jaeggi et al. [103], the threshold fluence for ablation increases and the penetration depth decreases in the ablation of copper and steel in air when increasing the pulse duration from 10 ps to 50 ps. Naturally, both effects negatively affect the ablation rate. If the pulse duration is further increased from the picosecond to the few nanosecond time regime, the threshold fluence for ablation becomes constant [104]. In addition, the ablation rate becomes independent from the optical penetration depths at these longer pulse durations due to the more thermal ablation mechanism [94]. Minor changes in the ablation rate can be assumed in the transition regime from picosecond to nanosecond pulse durations. However, the thermal nature of the laser ablation intensifies at even longer pulse durations. Heat losses get more dominant and the ablation rate is consequently reduced [105]. In addition, the lifetime of the laser-induced plasma and the pulse duration act on a comparable time scale for laser pulses longer than some ten or hundred nanoseconds, which intensifies a self-induced plasma shielding of single laser pulses.

**Figure 8:**
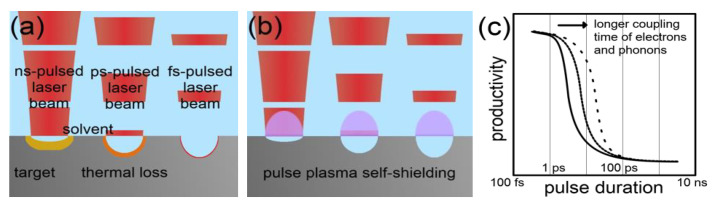
Illustration of the laser ablation of metals in liquids at different pulse durations neglecting non-linear effects. Thermal losses (**a**) and self-shielding by plasma (**b**) of pulses of different duration regimes. The diagram in (**c**) demonstrates how the coupling constant of electrons and phonons affects the NP productivity in LAL, depending on the laser pulse duration. A change of the coupling constant was assumed to have no influence on other material properties. In (**c**), the y-axis scales linearly. The trends shown in the diagram represent the interpretation of the combined results of the discussed literature in Section 2.2.1. In (**c**), the arrow and the less dense (dashed) line style indicate the effect of a longer coupling time of electrons and phonons of the target material on productivity. Reprinted with permission from [66].

In conclusion, an optimum in the pulse duration for achieving a maximized ablation rate in LAL exists. It is probably located in the order of single picoseconds [86]. As the productivity limitation at lower pulse durations stems from the optical breakdown of the liquid, the optimum of the pulse duration strongly depends on the laser intensity, wavelength and the liquid. Figure 8 illustrates the thermal loss and the pulse self-shielding by its induced plasma in dependence on the pulse duration regime. Furthermore, the dependence of the productivity on the pulse duration is shown qualitatively by excluding the optical breakdown of the liquid. The optical breakdown finds consideration in the following section on the laser intensity.

Laser Intensity: Since there is a tight connection between laser intensity, laser fluence and pulse duration, a differentiation between each contribution is difficult. The optical breakdown of the liquid environment, which may significantly reduce the ablation rate, is usually related to the laser intensity. Additionally, self-focusing of the laser beam may be induced by the optical Kerr effect and consequently increase the laser intensity. This may promote filamentation of the laser beam or an optical breakdown of the liquid. However, the intensity thresholds needed for the optical breakdown of water and the filament formation are comparable for femtosecond-pulsed laser radiation [106]. For picosecond pulses, the intensity threshold for filamentation is generally much higher than for the optical breakdown of water and becomes comparable only at low focusing angles [107]. Thus, filamentation plays no or only a minor role in LAL.

As described by Noack and Vogel [108], the laser-induced cascade of ionization must reach a specific density of free electrons to provoke the breakdown of the liquid in dependence on the pulse duration and wavelength. It is thus a competition of the recombination and ionization rate of free electrons, ions and atoms. Due to higher electron densities, plasmas induced by nanosecond laser pulses more effectively shield later irradiation than plasmas induced by picosecond pulses or long femtosecond pulses [109]. Additionally, one to two magnitudes of higher intensity thresholds for the optical breakdown of water are valid for femtosecond (10^12^ W/cm^2^) compared to nanosecond (10^10^ W/cm^2^) pulses.

The intensity threshold for the optical breakdown of the liquid environment further depends on the chemical nature and the purity of the liquid. In the case of linear hydrocarbons, Fujii et al. [110] found a decrease in the breakdown threshold for an increasing chain length or molecular weight using IR, nanosecond pulses. This is probably due to the increasing absorption cross-section of the hydrocarbons. The threshold intensities for the optical breakdown of different hydrocarbon aromatics at IR, nanosecond pulses are in the order of 10^12^ W/cm^2^ [111]. Kovalchuk et al. [112] demonstrated, temporally and spatially resolved, how particulate impurities in tap water and different alcohols act as seeds for the plasma formation in the laser-induced optical breakdown of the liquids. In addition, the presence of soluble species also significantly reduces the intensity threshold of a liquid for its breakdown [113,114]. It must be assumed that NPs act as solutes or impurities in this context. In addition, breakdown seeds may also be introduced into liquids by complexation of solvent molecules with dissolved gases [115].

**Figure 9:**
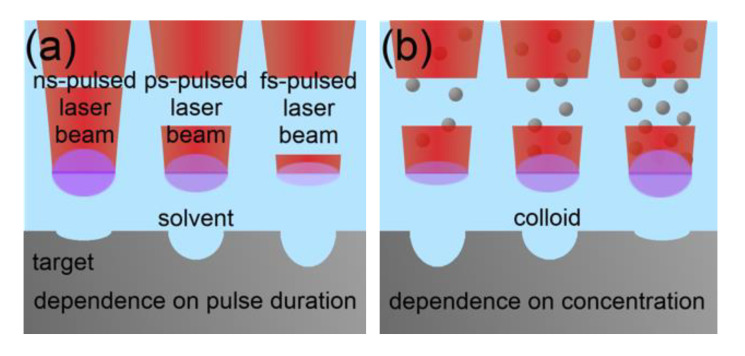
Illustration of the optical breakdown of the liquid during the laser ablation of metals in liquids at different intensities neglecting non-linear effects. Dependence of the occurrence of the optical breakdown on the laser pulse duration (**a**) and on the colloid concentration (**b**). Reprinted with permission from [66].

In conclusion, the optical breakdown of the liquid environment in absence of an ablation target must be excluded for the applied laser parameters in LAL. Thereby it needs to be considered that colloidal particles and dissolved species reduce the breakdown threshold. Whether an optical breakdown of the liquid occurs during LAL should therefore always be checked experimentally. Figure 9 illustrates how the optical breakdown of the liquid influences the productivity of LAL. Variations of the pulse duration and colloidal concentration were considered.

Spatial Inter-Pulse Distance: The interaction of laser pulses with laser-induced plasma, bubble and particle species limits the NP productivity in LAL. Whereas the shielding of a laser pulse by the self-induced plasma can only be reduced by using shorter pulse lengths, shielding effects induced by previous laser pulses can be reduced procedurally. The strategy is to spatially separate successive laser pulses by adjusting the interplay of the laser spot size, the scanning speed (relative movement between laser beam and target) and the pulse repetition rate to avoid shielding caused by the previous pulse [82,84,90,91,116].

Sattari et al. [84] found that the productivity of LAL can be increased by reducing the spatial overlap of successive laser pulses by increasing the scanning speed in the nanosecond-pulsed, IR laser ablation of Al_2_O_3_ in water. The authors achieved full pulse separation, and reached a maximum productivity at a specific interpulse distance. A linear decrease in productivity at higher distances followed. Wagener et al. [91] also observed an optimal interpulse distance in the picosecond-pulsed, Vis laser ablation of zinc in tetrahydrofuran. However, the decrease in productivity at longer interpulse distances without overlap was less pronounced compared to the results of the study of Sattari et al. [84]. This was probably due to the shorter pulse duration applied by Wagener et al. [91]. Streubel et al. [44] did not even observe a decrease in productivity at longer interpulse distances at an even shorter pulse duration. The thermal heating of the ablation target at longer pulses obviously influences the optimal interpulse distance.

**Figure 10:**
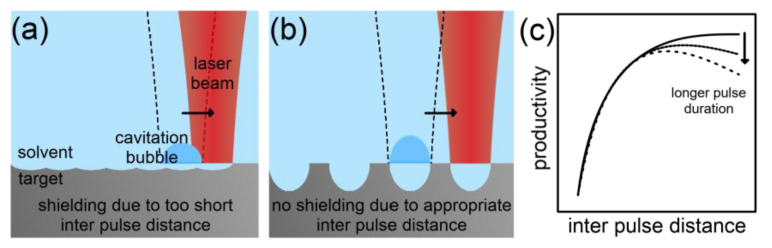
Illustration of a too short (**a**) and an appropriate (**b**) interpulse distance in the laser ablation of metals in liquids. The diagram in (**c**) demonstrates relative effects of different laser pulse durations on the dependence of the productivity on the inter-pulse distance. In (**c**), both axes scale linearly. The trends shown in the diagram represent the interpretation of the combined results of the discussed literature in Section 2.2.1. In (**c**), the arrow and the less dense line style indicate the effect of a longer laser pulse duration on productivity. Reprinted with permission from [66].

In summary, the productivity in LAL can be optimized by implementing a full spatial pulse separation. For laser pulses long enough to have a significant thermal impact on the ablation target, the productivity is maximized at an optimum interpulse distance. Figure 10 illustrates the effect of too short and appropriate interpulse distances on the productivity of LAL.

Liquid Environment: The relative motion [117,118], layer height [86,119,120] and physicochemical nature [66,71,121] of the liquid environment influence the productivity in LAL, as explained in the following.

A flowing liquid homogenizes the result of LAL compared to a static fluid environment [102,103]. As demonstrated by Streubel et al. [48], the productivity of LAL can be optimized by varying the flow velocity of the liquid. However, a dilution of the colloid must be accepted in parallel if LAL is applied continuously. The increase in productivity is probably caused by a faster transport of shielding species away from the ablation zone and an overall reduction of the concentration of shielding species. Consequently, the improvement of the productivity must saturate at a specific flow rate.

Interestingly, the height of the liquid layer on top of the ablation target also has an impact on the productivity. Al-Mamun et al. [86] observed a maximized ablation rate at a minimized layer height of 2 mm and Jiang et al. [122] found an optimum height at 1.2 mm. Both research groups assumed a confinement effect as a reason for a generally higher productivity at lower liquid layer heights. For example, shock waves generated in the ablation process could be reflected back to the target from the liquid–air interface. In addition, the experimental implementation must also be considered. Especially for long ablation times and batch setups, the exponential absorption of the laser radiation by the nanoparticle colloid, described by the Beer–Lambert law, will be much stronger at high liquid levels. Menéndez-Manjón et al. [44] further pointed out the importance of the compensation of the change in beam propagation induced by the refraction at the air-liquid interface, when changing the liquid layer height. The authors also describe the productivity-limiting effect of explosive vaporization of the liquid at a critical minimum of the liquid layer height. This effect may have led to the optimum liquid layer height observed by Jiang et al. [119].

The impact of the physicochemical nature of the liquid on the productivity in LAL is more complex. Baladi and Mamoory [121] investigated the ablation rate in the nanosecond-pulsed, IR laser ablation of aluminum in ethanol, acetone and EG (ethylene glycol) at comparable laser fluences. They found similar ablation rates for ethanol and acetone. However, the ablation rate during LAL in EG was lowered by more than 90%. Cristoforetti et al. [87] observed comparable productivities in the nanosecond-pulsed, IR laser ablation of palladium in ethanol and 2-propanol, but a productivity reduction in acetone (by 15%), water (by 20%) and toluene and n-hexane (by 85%). The reasons for those differences in productivity remained unclear.

Kalus et al. [120] also observed significantly different ablation rates during LAL (nanosecond-pulsed, IR) in water compared to mono-, di- and tri-EG. The authors could correlate the reduced productivity to intensified shielding of the laser beam by persistent bubbles. They found that the size and dwell time of the bubbles depends on the viscosity of the liquid. Consequently, a higher viscosity promotes shielding and reduces the ablation rate. However, the low-viscous liquid ethyl acetate did not fit the observed trend. There must be at least one other factor such as the vapor pressure or the specific heat of vaporization considered to make the shielding by persistent bubbles more predictive.

Another study on the productivity of LAL in different liquids was conducted by ablating iron using IR, femtosecond laser pulses. Interestingly, the authors observed an optimum in the energy-related productivity for all tested liquids (water, methanol, ethanol, acetone and toluene) at a similar fluence but with different ablation rates. Again, the lowest ablation rate was observed in toluene, 85% lower than in water. The low applied repetition rate of 1 Hz combined with a low liquid layer of 4 mm should have minimized shielding effects by persistent bubbles. In addition, no significant difference was found in the ablation-effective spot diameter and the amount of reflected laser energy. However, the plasma radiation in different liquids qualitatively mirrored the productivity. The question for the productivity-lowering liquid parameter remained unanswered, but differences in the laser penetration depth into the ablation target, as assumed by the authors, were unlikely to take place at the short pulse duration of 35 fs.

**Figure 11:**
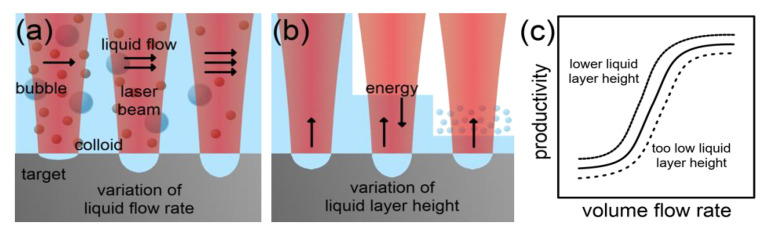
Illustration of the effect of the liquid flow rate (**a**) and of the liquid layer height (**b**) on the productivity in the laser ablation of metals in liquids. The diagram in (**c**) demonstrates relative effects of different liquid layer heights on the dependence of the productivity on the volume flow rate. In (**c**), both axes scale linearly. The trends shown in the diagram represent the interpretation of the combined results of the discussed literature in Section 2.2.1. In (**c**), the dashed line shows the effect of lower liquid layer height on the productivity, and the dashed line shows the effect of a too low liquid layer height compared to the solid line, respectively. Reprinted with permission from [66].

In summary, the productivity of LAL can be improved by applying a liquid flow, by choosing a low liquid level, and by performing LAL in water. In specific cases, water undesirably alters the properties of the NPs, for instance, by oxidation. Low-viscous organic solvents like ethanol or acetone represent the best alternatives in such a case. Figure 11 illustrates the effect of the flow velocity and the liquid layer height on the productivity of LAL.

Target Geometry: There are three dimensions to vary on the geometry of a bulk ablation target. By reducing single dimensions of a bulk to sizes comparable to the laser spot, the available target geometries include foils, wires, and particles. An impact on the ablation rate is discussed below.

Scaramuzza et al. [123] investigated the impact of the dimensional reduction of the ablation target on the productivity. The authors found a decrease in the productivity when ablating gold foils with thicknesses less than 0.1 mm covered with an aqueous saline solution. Nanosecond, IR laser pulses with a fluence of 4.3 J/cm^2^ were used. Additionally, debris of gold were present in colloids synthesized from foils with thicknesses between 100 nm and 0.1 mm, which led to an overestimated ablation rate.

The reduction of another dimension of the ablation target leads to LAL of wires. Messina et al. [124] achieved ablation rates, which were by a factor of 15 higher compared to a bulk target in the nanosecond-pulsed laser ablation of silver wires in water at a fluence of 1.5 J/cm^2^. An optimal wire diameter of 0.75 mm existed. The laser beam diameter was 3 mm. A confinement of heat in the wire and differing cavitation bubble dynamics were named to cause the improved productivity. The method of how the confinement of heat should affect the wire temperature was calculated by Scaramuzza et al. [123]. The dynamics of the cavitation bubble were investigated by De Giacomo et al. [122] in a comparative study on bulk and wire ablation.

A recent study of Kohsakowski et al. [90] reported significantly lower differences in LAL of silver wires and bulk targets. The wire ablation was more productive by a factor of 1.47. This contradictory result compared to the results of Messina et al. [124] at comparable laser parameters were mainly due to optimized process parameters in the bulk ablation by Kohsakowski et al. [90].

**Figure 12:**
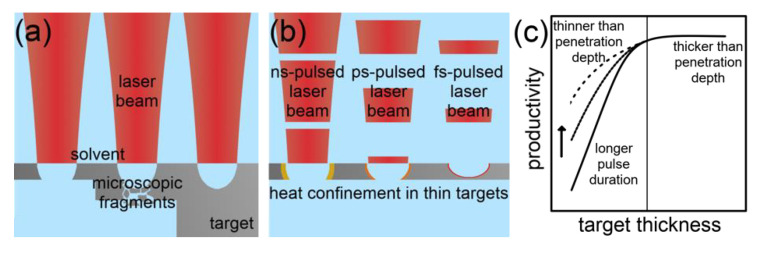
Illustration of the effect of the target thickness in the laser ablation of metals in liquids (**a**) and the influence of the laser pulse duration on the heat confinement in thin targets (**b**). The diagram in (**c**) demonstrates relative effects of different laser pulse duration on the dependence of the productivity on the ablation target thickness. In (**c**), both axes scale linearly. The trends shown in the diagram represent the interpretation of the combined results of the discussed literature in Section 2.2.1. In (**c**), the arrow and the less dense line style indicate the effect of longer pulse duration on productivity. In (**c**), the vertical solid line in the center of the diagram marks the penetration depths of the laser pulse into the target material. Reprinted with permission from [66].

In conclusion, a higher productivity of LAL can be accessed when ablating macroscopic metal targets compared to thin foils. However, by laser ablating a wire, the highest productivity in nanosecond-pulsed metal ablation should be achievable due to heat confinement. An optimal wire diameter exists, probably in dependence on the laser parameters. Heat confinement in the ablation target plays a minor role in ultrashort-pulsed laser ablation of metals, making wire ablation probably less advantageous here. Figure 12 illustrates the described effects of this section.

Critical View on Possibilities of Productivity Improvement: Obviously, the amount of productivity-effective process parameters and variables in LAL is extensive and hard to cover due to the variety and the existing cross-dependencies between the parameters and variables. In addition, the relevant literature does not provide the data required for a productivity evaluation in a full-range parameter variation, not even for a single laser system. Furthermore, important studies like that of Riabinina et al. [88] on the impact of the pulse duration on the productivity investigated only one material and liquid environment, and neither scanning of the laser beam nor a liquid flow were applied.

Other important aspects are the limitations given by the equipment and the desired product. In case specific oxidation states or size distributions of NPs are required, laser ablation probably needs to be performed at specific laser parameters and in a particular liquid. Both may lower the ablation rate. However, there is an optimum set of laser parameters of a specific laser system for each combination of ablation material and liquid environment. Ideally, this set is evaluated experimentally in each case productivity plays a major role.

### 2.3. LAL-Based Techniques for Nanomaterials Synthesis and Processing

In the previous sections, the fundamental mechanisms involved in the LAL process are described considering the effects of laser parameters, and how the active species act differently in plasma, cavitation bubbles and droplets as a function of the ablation conditions. We have seen that the liquid is the second most important parameter after the laser source in LAL. Water and common organic solvents, including alcohol, acetone and sodium dodecyl sulfate (SDS) have been used widely in LAL. Moreover, small biomolecules such as aqueous oligonucleotide solutions, polymers, liquid nitrogen, supercritical carbon dioxide and liquid helium at very low temperatures have been employed as unique liquids to synthesize nanocrystals and fabricate nanostructures [125].

Recently, important progress to optimize NP production has been also attained following the introduction of field-assisted LAL. Importantly, the experiments have proven that the morphology, composition and structure of LAL-generated nanomaterials can be readily controlled by changing the external environment. Various field-assisted LAL techniques have been developed, such as temperature field-assisted LAL [126], electric field-assisted laser ablation in liquid (EFLAL) [127], Magnetic Field-assisted Laser Ablation in Liquid (MFLAL) [128] and electrochemistry-assisted laser ablation in liquid (ECLAL) [129].

Polyoxometalate (POM) materials, consisting of at least two (usually three or more) transition metal oxyanions that are linked together by shared oxygen atoms to form a large, closed three-dimensional framework, were successfully synthesized by ECLAL. POM nanostructures are attractive because of their high catalytic activity in oil refining, their favorable emission properties and suitability for energy storage applications [130]. With respect to many other synthesis techniques, ECLAL advantages are: (i) clean preparation of nanostructures because of its simple starting materials and limited byproduct formation; (ii) an ambient environment without extreme temperature and pressure conditions; (iii) flexibility to design various nanostructures by combining specific solid targets, electrodes and liquids. Liu et al. [131] first prepared POMs by a one-step route using molybdenum as a solid target, copper as electrodes, and deionized water as the solvent. Two copper electrodes were placed between the two facing sides of a chamber. A steady electric field with a voltage of 20 V was produced by a DC power source. The XRD pattern of the as-synthesized nanostructure indicated that it was a monoclinic phase of crystalline lindgrenite (Cu_3_(OH)_2_(MoO_4_)_2_). No signatures of amorphous molybdenum, copper compound or any other oxide phase were found in the XRD pattern, indicating that the synthesized sample is highly pure and crystalline. It emerges that different products should be obtained combining targets and electrodes of different nature. So, Liang and co-workers used three different kinds of combinations, vanadium (target) + copper (electrode) [132], vanadium (target) + silver (electrode) [133] and molybdenum (target) + zinc (electrode) [6], to synthesize ZnMoO_4_ micro- and nanoplates and rods, Cu_3_(OH)_2_V_2_O_7_·2H_2_O flower-like nanostructures, and Ag_2_V_4_O_11_ brush-like nanostructures. It is worth noticing that in ECLAL, the targets and electrodes used react. This does not occur in EFLAL.

For instance, Ismail et al. [134] reported the production of Bi_2_O_3_ NPs by the pulsed laser ablation in water under the effect of the electric field (EFLAL). They found that applying the electric field during laser ablation led to the increase of the Bi_2_O_3_ particle size. Sapkota et al. [135] used an excimer laser (351 nm) to ablate a solid tin target immersed in water in the presence of an external electric field, while Jumaa et al. [136] reported the synthesis of gold NPs by the pulsed Nd:YAG (1064 nm) laser ablation in deionized water mainly to study the effect of the electric field on the antibacterial activity of these particles. Al-Haddad et al. [137] synthesized Au NPs by pulsed laser (Nd:YAG, *λ* = 1064 nm), ablation of a gold target immersed in deionized water by 300 mJ of laser energy, while a DC electrical field was applied above the target with adjusted voltage. The influence of the applied electric field on the formation of platinum NPs by laser (Nd:YAG, *λ* = 1064 nm) ablation technique was investigated by Moniri et al. [138]. Furthermore, laser ablation of germanium in liquids with static, externally applied electric fields is discussed in [139]. The authors found that ablation in water results in spherical NPs and filaments while ablation in ethanol favors the synthesis of spherical and spindle-shaped NPs. The latter resulted from ablation only with an electric field strength of 9.5 V/cm, and the target being immersed in ethanol. A clear correlation between decreasing spherical particle size and increasing externally applied electric field was found. Similarly, Liu et al. [127], adopting EFLAL, with the second harmonic of a nanosecond-pulsed, Q-switched Nd:YAG laser-prepared GeO_2_ micro and nanocubes with high-index facets and a kind of GeO_2_ micro and nanospindles. This approach avoids the substantial quantities of capping agents that are attached to the surface of the GeO_2_ nanostructures, as observed on germanium oxide nanomaterials prepared by sol-gel reaction. The synthesis of GeO_2_ nanocubes and nanospindles is ascribed to the interaction between the laser beam and the external electric field. In the process, first the Ge plasma is expected to form because of the high temperature and pressure induced by laser. Meanwhile, the active O and/or OH species that originate from the decomposition of water would surround the plasma and react with it to form GeO_2_ NPs. X-ray diffraction (XRD) and selected-area electron diffraction (SAED) patterns of the products revealed that the preferred {1011} plane of GeO_2_ was dominant. This suggests that the applied electric field helped to stabilize the growth of {1011} planes, while hindering the growth of planes perpendicular to such planes. In this case, the six {1011} planes readily formed into the nanocube morphology. As the applied electric field became stronger, the growth of the {1011} plane would be elongated along the [1] direction, which results in the formation of spindle-like morphology. Thus, the electric field plays an important role in governing NP shape.

On the basis of the above-described evidence, it emerges that EFLAL extends the traditional LAL approach, allowing a better control of the morphology, size, chemical composition and structure of the metal-based nanostructures as well as an increase of the NP concentration. This is explained taking into account that: (1) electric field is believed to transport the charged particles produced in the plasma plume at the electrodes, in a process that resembles electrophoresis; (2) EF allows an efficient removal of the bubbles formed both in the volume of the liquid and on the target. The delivery of laser pulses onto the target is more effective and so the ablation rate in liquid is higher by a factor of three (generally, about 300 mg/30 min) compared to ablation without EF (about 100 mg/30 min).

**Figure 13:**
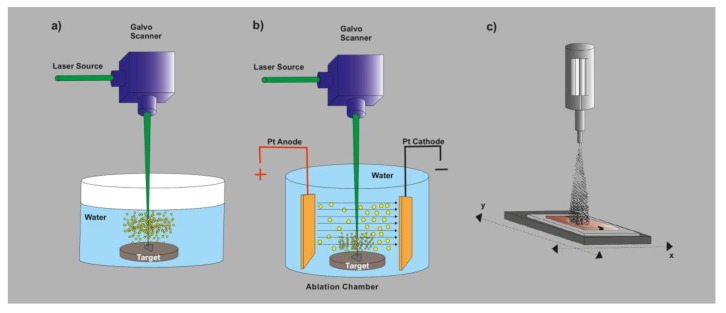
Schematic of LAL (**a**) and EFLAL setups (**b**); ultrasonic atomizer setup (**c**).

The EF role on Mo-based nanostructures is also shown by Spadaro and Fazio et al. [140,141] that deal with the synthesis of Mo oxide nanocolloids using a focused picosecond-pulsed laser beam (*λ* = 532 nm, repetition rate = 100 kHz, pulse width = 6–8 ps). A molybdenum target was ablated in water and the effect of an external EF (the estimated electric field is 20 V/cm, the Pt electrode distance fixed at 5 cm) applied during the ablation process, has been exploited in order to check for modifications of the NP surface morphology and surface chemical bonding configurations. The obtained colloids were sprayed by means of an ultrasonic atomizer on glass substrates to carry out the chemical-physical characterizations (see Figure 13). A selective tuning of Mo–O chemical bonding configurations and a suitable control of NP size distributions were achieved by varying the water temperature and by applying the external EF. Two types of nanocolloids were prepared: one with a spherical shape, size less than 50 nm and a dominant MoO_2_ surface chemical configuration, and another with an oblong structure, where the oxygen deficient MoO_3_ phase increases at the expense of the MoO_2_ one. Both nanocolloids induce metal redox and antioxidant properties which strongly depend on the Mo–O surface chemical bonding configurations and NP morphology.

Overall, the main interesting evidences are: (i) the possibility of tuning NP surface composition and surface/volume ratio directly in the production phase, without any post-synthesis treatment, (ii) the ability to modulate their bio-properties as the production of Reactive Oxygen Species (ROS) and antioxidant capability, which affect cell-signaling mechanisms. Ultimately, the possibility to tune the properties of Mo-based nanostructures is to be considered useful for future innovative biomedical applications to identify potential anticancer complexes acting through ROS production or redox dependent mechanisms.

In the next sections, we draw the reader’s attention to a report with specific details on promising applications of LAL nanostructures (mainly plasmonic metal-based NPs) for biotechnology applications and for organic pollutants degradation. Remarkably, these fields were not covered, to the best of our knowledge, in previous reviews that were mostly dedicated to catalysis.

In addition, based on our interactions with industrial partners and pharmaceutical companies, at present we believe that the main difficulty is cultural. The technicians of these companies are mainly chemists by training, and therefore have a preference for chemical techniques that they feel closer to their culture. A simple explanation, but a difficult barrier to overcome.

## 3. Promising Applications of LAL Nanostructures for Biotechnology Applications and for Organic Pollutants Degradation

### 3.1. Plasmonic Properties of Metal Nanoparticles and Plasmon Sensitivity

Plasmonics involves the control of light at the nanoscale by using surface plasmons. Localized surface plasmon resonance (LSPR), which imparts unique optical properties to metal nanostructures, involves the collective and coherent oscillation of dielectrically confined conduction electrons on the surface of metal nanostructures under the effect of electromagnetic fields [142,143].

Owing to the unique combination of physical and chemical properties, such as large absorption and scattering cross-section, high sensitivity to local dielectric environment and enhanced electric field at the surface, plasmonic nanostructures are emerging as an important class of materials for various optical sensing applications. Plasmonic systems in particular have been studied extensively due to their ability to confine light below the diffraction limit, which greatly enhances their sensitivity compared to conventional approaches [144]. The oscillating electric field of an electromagnetic wave causes the formation of dipoles and multipoles in metallic NPs. Such multipoles depend on the size and shape of the NP in a way predictable by solving the Maxwell equations at the interface between the metal and the external medium. For noble metals, the plasmonic response of the NP falls into the UV-vis-NIR frequency range and it is significantly observed by considering the absorption and scattering cross sections. In a classical transmittance experiment the overall extinction effect (the sum of scattering and absorption) can be measured. If we restrict our consideration to a spherical NP with a size much smaller so that the impinging radiation wavelength scattering can be neglected (quasi static approximation) and a simple dipole is sufficient to account for the plasmonic behavior:

(10) μ=αE

where *α* is the electric polarizability and *E* is the electric field. In this case the polarizability is expressed as:

(11) $$\alpha =4\pi \varepsilon_{0}R^{3}\frac{\varepsilon -\varepsilon_{m}}{\varepsilon +2\varepsilon_{m}}$$

where *ε* is the dielectric function [$\varepsilon_{1}\left(\omega \right)+i\varepsilon_{2}\left(\omega \right)$] and *R* the radius of the NP. The resonance condition is achieved for maximum polarizability, which happens for *ε = −2ε_m_*. It is thus clear that plasmon resonance depends on the metal through its size and dielectric function and on the medium through its dielectric constant *ε_m_* (and thus the refractive index *n*). Much more complex is the situation in all those cases where the NP has a shape far from spherical-like and proper simulations about the plasmonic response should be conducted to interpret the response [142,144,145].

**Figure 14:**
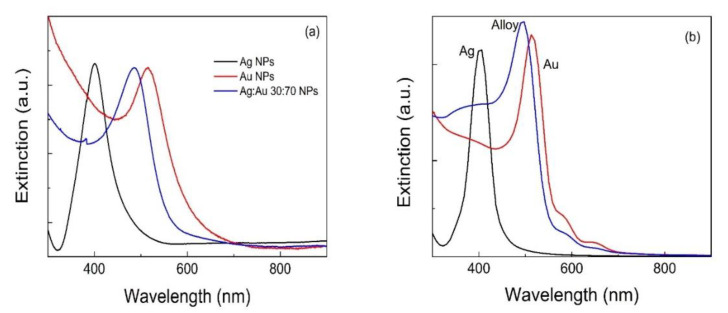
Absorption spectra of Ag, Ag:Au and Ag NP colloids produced from: (**a**) laser ablation in liquids of Ag, Ag:Au alloy and Au targets, respectively; (**b**) a Boundary Element Method (BEM) simulation.

Laser ablation of metallic targets permits the formation of noble metal NPs in a range of solvents, including water and alcohols. Among noble metals, gold has been the most intensely studied, followed by silver. Both NPs can be produced as stable suspensions by LAL with diameters in the range 10–30 nm. Being different metals, they have different dielectric functions allowing plasmon resonances in different spectral regions. Indeed, Ag NPs have their typical plasmon resonance just below 400 nm, which instead lies around 510–530 nm for Au NPs. Despite plasmon resonance depending on particle size, a tuning across a broad range of the spectrum is not achievable just by adjusting spherical particle size. One way to overcome the issue is by alloying [145,146]. Figure 14a shows that even Au/Ag colloidal NP alloys can be grown if the ablation is performed using an alloy target in water [147]. Here, we refer to a gold 70% molar concentration with respect to silver, but any result can be obtained by tuning the concentration, thanks to the total miscibility of the two metals. This ensures the possibility to tune the plasmon resonance from that of silver (400 nm) to that of gold (520 nm) continuously, opening the way to a fine plasmonic response. Alternatively, a very similar result can be obtained by pulsed laser irradiation of a previously formed Au/Ag colloidal mixture [147]. While in the former case the plasmon resonance tuning can be achieved only by choosing a different alloy target for every composition, in the latter case, the colloidal mixture can be prepared in any metal ratio from pure metal colloids, which is a clear advantage. Figure 14b reports a range of simulations conducted using a Boundary Element Method (BEM) developed by A. Trügler [148]. In this case a dielectric environment (water) where bodies with homogeneous and isotropic dielectric functions are separated by abrupt interfaces is assumed, thus solving Maxwell equations using the boundary conditions at the particle boundaries. As for the dielectric function of the considered Au/Ag alloy, a weighted average method was used, in which the dielectric function of the alloy is defined as:

*ε*_alloy_(*ω*) = *x ε*_Au_(*ω*) + (1 − *x*) *ε*_Ag_(*ω*) (12)

where *x* denotes the Au molar fraction in the Au−Ag alloy [149].

The simulation results shown in Figure 14b agree with the experimental results concerning the plasmon resonance position. The smaller width of the simulated extinction spectra is attributed to the difficulty to set an appropriate damping constant. The same BEM method predicts that as an Au NP grows larger, the plasmon resonance slightly red-shifts and broadens. However, in order to be able to tune the plasmon resonance of a metallic NP, playing with the size is not enough to explore a broad range [143,150]. It has to be noted that Equation (1) is strictly valid only for spherical particles, while for particles with different shapes, the geometry must be taken into account and a plasmon resonance shift can be expected. Indeed, beside the aforementioned alloying, a shape change is another effective strategy to tune the plasmon resonance. This was observed experimentally in gold nanorods [151,152], gold nanostars [153] and silver nanoplates [154,155].

**Figure 15:**
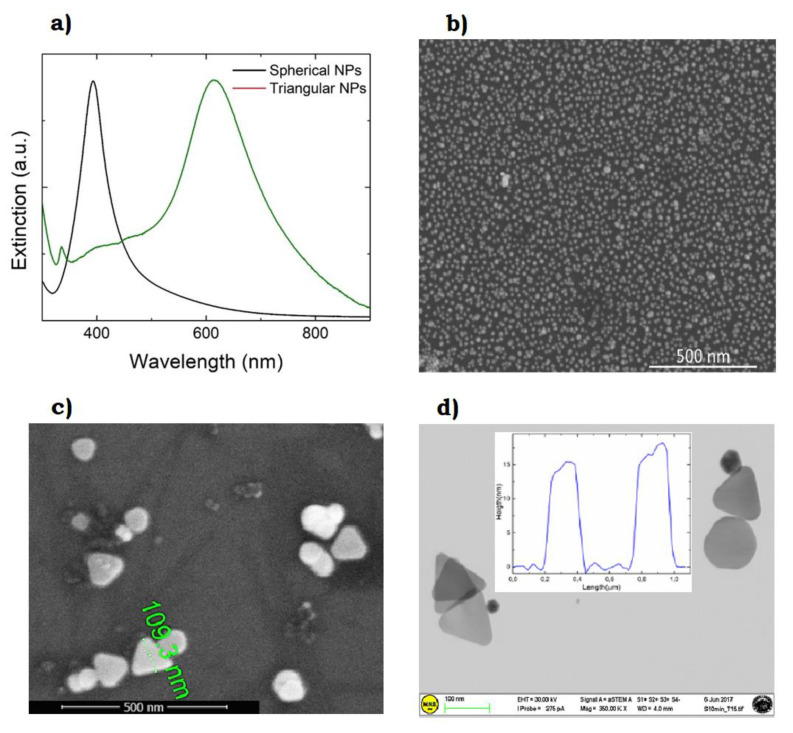
Optical absorption spectra of spherical vs triangular silver NPs (**a**); SEM image of spherical NPs (**b**) and of triangular nanoparticles (**c**) TEM image and in the inset AFM profiles of triangular NPs (**d**). Reprinted from Ref. [156], Copyright (2019), with permission from Elsevier.

As an example, Figure 15a shows how the typical plasmon resonance observed around 400 nm for spherical Ag NPs is red-shifted to around 600 nm as the particles are transformed to flat triangular nanoplates. Spherical NPs were obtained by LAL in water with citrate as capping agent, using a 1064 Nd:YAG-pulsed laser. These show a single plasmon resonance feature at 393 nm, indicating particles in the region of 10 nm, as can also be observed by SEM in Figure 15b. When such particles are irradiated with white light, in association with H_2_O_2_ addition, a dramatic spectral change occurs, with the formation of two main features [156]. One feature is red-shifted to ~600 nm and represents the longitudinal in-plane dipole mode [157]. The other important feature, despite its low intensity, is the out-of-plane quadrupole mode, at 335 nm, which is an indication of anisotropy in the structures [157]. Indeed, spherical particles are converted into flat triangular ones, sized around 100 nm and thick about 10–20 nm, as shown by scanning electron microscopy images and atomic force microscopy profiles in Figure 15c,d.

Such transformation is typically achieved using a process named “seed-mediated growth”, which involves the chemical synthesis of Ag NPs starting from an Ag^+^ salt and reducing agents as a first step, followed by a further chemical process to transform initial spherical particles, or “seeds”, into triangular nanoplates [158,159,160]. However, the use of LAL for the production of the seeds, coupled with light irradiation and H_2_O_2_ addition, has the great advantage of being an environment-friendly process that does not involve the use of strong reducing agents or harmful chemicals [156,161,162]. Moreover, LAL is a very versatile tool where virtually any material can be produced in any liquid without any other chemical involved. This allows for a wide freedom in the use of a variety of capping agents which can be chosen depending on the final application or process needs. Indeed, citrate is an excellent example as, within the process described above, it is used as both a capping agent to stabilize the particles in water, as a driver for growth and reshaping and as a mild reducing agent. The above mentioned dependence of the plasmon resonance on the refractive index of the medium can be exploited for optical sensing applications [163], even at a single molecule level [164,165], as it allows the detection of molecules dissolved in the colloidal phase or adsorbed on the surface, as long as they cause a variation of the refractive index of the medium [166,167]. The key property is the variation of the plasmon resonance relative to the refractive index change, or plasmon sensitivity, which can thus be defined as:

(13) $$S=\frac{\Delta \lambda }{\Delta n}$$

where Δ*λ* is the plasmon resonance variation in wavelength associated to the refractive index variation Δ*n*. *S* is typically expressed in terms of nm/refractive index unit (RIU). *S* can be easily measured by performing a relatively simple experiment in which a small aliquot of colloid solution is mixed with a sucrose solution at a set concentration, in a 20–70% range, where a direct relationship between sucrose concentration and refractive index exists [168].

**Figure 16:**
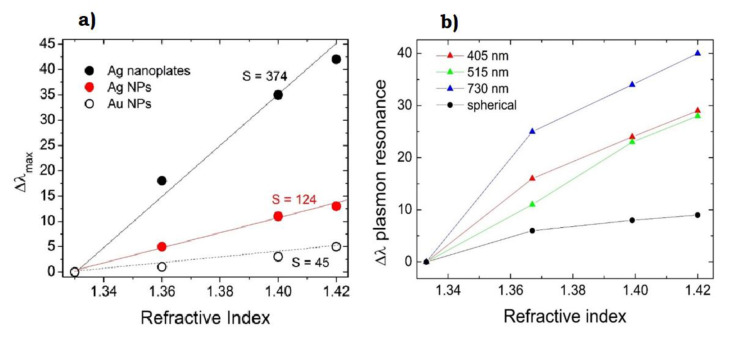
Plasmon sensitivity measurements from gold and silver spherical nanoparticles, obtained by laser ablation in water, and silver triangular nanoplates obtained by white light irradiation of the same spherical NPs (**a**); plasmon sensitivity measurements from triangular NPs obtained from monochromatic light irradiation of spherical NPs (also shown) obtained by laser ablation (**b**). Figure 16a reprinted from Ref. [156], Copyright (2019), with permission from Elsevier. Figure 16b reprinted from Ref. [170], with the permission of The Royal Society of Chemistry.

Figure 16a shows an example of plasmon sensitivity measurements for Au and Ag spherical NPs produced by LAL in water. Ag NPs show higher sensitivity (125 nm/RIU) than Au NPs (45 nm/RIU) and are thus more widely investigated for these type of applications. However, when spherical NPs are converted into nanoplates, the plasmon sensitivity shows a roughly threefold increase [161,169], as shown in Figure 16a. Plasmon sensitivity can be further increased if the Ag NP colloid is irradiated with monochromatic light instead of white light [170]. Indeed, irradiating at 730 nm causes the formation of particles with *S* = 460 nm/RIU [170] (Figure 16b). On the other hand, other irradiation wavelengths yield NPs with lower sensitivity, hence the wavelength of choice appears a key factor in enhancing plasmon sensitivity.

This plasmon sensitivity behavior has the potential for the development of sensing systems where the presence of an analyte in solution could be detected by simple optical measurements, provided they induce a change in the refractive index. Selectivity in this case remains a challenge that researchers have to solve, for example through specific functionalization of the NPs.

### 3.2. Plasmonic Nanocolloids for Biotechnology Applications

Colloidal solutions of plasmonic NPs synthesized by laser ablation in solution (LAL) are key materials for biotechnology applications. We discuss some of our contributions to recall such applications. NPs are synthesized without stabilizing molecules, in order to avoid any associated biocompatibility problem. Furthermore, the naked surface of these NPs can be easily functionalized, for example with molecules for active cell targeting [171,172,173], for sensing and diagnosis [174,175,176], for drug monitoring [177] and also for therapies, as in the case of photothermal therapy [171,175]. Using multiple functionalizations, theranostic nanosystems, namely nanosystems useful for both therapy and diagnosis, can also be obtained.

Thanks to their chemical stability gold nanoparticles (AuNPs) are very frequently used in comparison to other metallic NPs, such as Ag NPs, which cause toxic effects due to ion solubilization. The presence of localized surface plasmons (LSPs) is an interesting property of this type of NPs that can be exploited for many applications. LSPs arise from collective oscillations of the free electrons of metal nanoparticles and show resonances in the visible and near infrared spectral range, depending on shape, dimensions and aggregation of the nanostructures, with very large extinction cross sections if compared to organic dyes [154]. LSP resonances (LSPR) depend also on the particle surrounding (in particular by its refractive index) and sensors can be developed on such a dependence. Moreover, excitation of the LSPR amplifies the incoming electromagnetic field at the NP surface by some orders of magnitudes within a few nm [178]. This amplification made possible the exploitation of Raman spectroscopy in the field of sensing technology with the technique called Surface Enhanced Raman Scattering (SERS). In this case, for molecules near the surface of plasmonic NPs, one can obtain amplifications of the vibrational Raman spectrum of up to 10 orders of magnitude and even more in favorable conditions [178].

NPs synthesized by LAL are of particular interest because of their clean surface, which allows functional molecules to reach easily the NP surface [179,180]. Antigen cell targeting [171,172,173,180,181,182], direct or indirect analytical sensors [177,179,183] and multimodal contrast agent in vivo [182] are examples of recent results obtained in our laboratories with plasmonic nanostructures for biotechnology applications.

The identification of antigens expressed by cells allows active targeting of individual cells, which is relevant in the early stage recognition of cancer cells. We developed a methodology for detecting cancer cells based on sub-nanomolar concentration of nanocolloids functionalized with monoclonal antibodies [147,148] or with engineered targeting peptides [172,180,181,182,183]. SERS labels, namely molecules showing huge SERS signals when coupled to plasmonic NPs, were used on the nanostructures to identify active targeting events using the presence of SERS signals on individual cells incubated with the nanostructures. SERS signals are vibrational signals, which are easily recognized and cannot be confused with other signals, like in the case of fluorescent signals usually exploited for this purpose. Our protocol is efficient because the naked particles, synthesized by laser ablation, are functionalized simply by mixing the colloidal solution of Au NPs with the solutions of the targeting molecules, without the need to control the exchange with stabilizing molecules used in other NP preparations. Molecules and also antibodies are thiolated to assure a strong link with the Au NPs. Strong SERS signals are obtained by controlling the aggregations of NPs, which create the hot spots where molecules show very intense SERS signals.

Incubations of the functionalized nanostructures were obtained with cancer cells overexpressing, or not, the target antigen. For the active targeting of prostatic cancer cells, nanostructures functionalized with antibodies for prostatic specific membrane antigen (PSMA) and prostate stem cell antigen (PSCA) antigens were considered [178,180]. Sensitivity (correct targeting among positive cells) and specificity (absence of targeting among negative cells) of the order of 90% were obtained showing the efficiency of the prepared nanostructures [180].

Monoclonal antibodies can be, however, immunogenic and usually their cost is very high. Small molecules like peptides are targeting units that do not show the problem of immunogenicity and their cost is much lower. In this case, however, the affinity for specific antigens is low. We have prepared nanostructures with the following peptides: GE11, which targets epidermal growth factor receptors (EGFRs) expressed in different types of tumors [172,181], RGD for v3 integrin, an adhesion receptor found in tumors at metastatic level [182], targeting and PreS1 for SerpinB3, a protein overexpressed by liver tumor cells, targeting [180].

**Figure 17:**
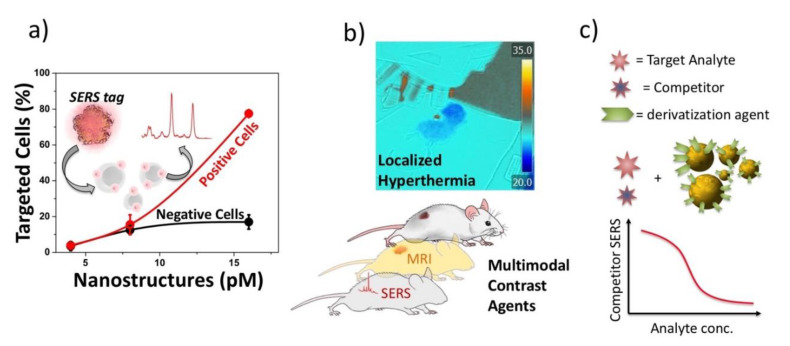
Nanoparticles obtained by LAL are particularly versatile for biotechnology applications. (**a**) active antigen targeting of cells overexpressing or not the antigen using Surface Enhanced Raman Scattering (SERS) signals; (**b**) multimodal contrast agents and hyperthermal experiments; (**c**) therapeutic drug monitoring with a competitive SERS protocol.

The results show the need to engineer the peptides for the NP functionalization. A long polyethylene glycol (PEG) chain (3000 molecular weight) and a short-charged lysine sequence linked to the active peptide are required to obtain high targeting efficiencies that, in particular for specificity, are found to be also better than those obtained with a specific monoclonal antibody already used in clinic. The results suggest that the large number of peptides (thousands per particle) per NP overcome the problem of the small affinity of the single peptide, exploiting the avidity of an ensemble of targeting units on a single nanostructure. With this approach, sensitivity and specificity larger than 80–90% are obtained [171,182] (see Figure 17a). Models for the arrangement of the targeting units on the Au NP surface, obtained with Molecular Dynamics calculations, show that the presence of the short lysine charged sequence is strategic for obtaining the appropriate exposition of the targeting peptide over the PEG chains, collapsed over the NP surface [172,182].

SERS signals, as a new approach for the detection of targeting events are much more efficient than the usual fluorescence signals and allow very easy multiplexing measurements because of the very narrow bands that can be recorded for vibrational SERS spectra with respect to the very large bands observed for fluorescence spectra [175]. SERS signals are particularly useful when used for in vivo studies where autofluorescence of tissue and other biological components hamper the detection of low intensity fluorescence signals. This is not the problem of SERS signals in particular when they derive from SERS labels with known spectra. After inoculation of nanostructures with SERS signals into mice, we were able to easily find SERS signals in their organs where nanostructures accumulated, or in a tumor, whose margins can be easily obtained with SERS signals. An example is the targeting of SerpinB3 antigen, expressed by liver tumor cells in a mouse which overexpressed this protein, using nanostructures functionalized with the peptide PreS1. The targeting activity was first evidenced in in vitro studies and, when applied to animal trials, the targeted organs reported bright SERS signals with a complete clearance in 6 h [180]. Another example was obtained with nanostructures constructed as multimodal contrast agents. In particular we obtained a nanostructured system in which a SERS label and an MRI contrast agent based on Gd3+ were present. MRI images of a tumor were obtained for mice injected with the nanostructures together with SERS images, which easily showed the margin of the tumor [182] (see Figure 17b).

These experiments also made it possible to use NPs for photothermal therapy of the tumor, evidencing an increase of the tumor far field measured temperature, after 10 min of irradiation, by 8 degrees which is considered sufficient to start the apoptosis mechanism for the tumor cells. In vitro experiments also showed that few minutes of irradiation allowed killing about 98% of irradiated cells, which were targeted by the functionalized NPs [171].

SERS signals are usually considered not appropriate for quantitative evaluations because of uncertainty in the reproducibility of the SERS substrate with the present hot spots. We have participated in a very recent collaboration, in which NPs, produced by laser ablation at our lab, were tested in 15 laboratories around Europe in an experiment coordinated by the University of Trieste within a COST action (Raman4clinics). The results were very satisfactory and show that, in particular with the Au NPs, a linear relationship between signals and analyte concentration was obtained with appropriate reproducibility and trueness [179].

Furthermore, we found that a quantitative approach can be obtained in an application like Therapeutic drug monitoring (TDM) also with drugs spiked in plasma, which simulates a real situation. Plasma is a difficult matrix due to the large concentration of proteins, which interfere with the availability of hot spots of the nanostructures for the SERS spectra. It is worth to recall the protocol used for the TDM in the case of Erlotinib, a therapeutic drug for cancer cells overexpressing EGFR [153]. A SERS measurement requires that the molecule to be revealed shows strong SERS signals, which is usually observed when a resonance of the exciting laser line with the molecular excitations is present, obtaining the so-called surface enhanced resonant Raman scattering (SERRS). Since this is not always possible for the molecules to be revealed, we found that a competitive approach is more efficient. This is the case of the Erlotinib molecule, which absorbs in the UV spectral region, far from the laser excitations in the visible, or near infrared spectral region where the LSPR lie. In this case, a molecule with strong SERRS signals is used, in competition with the molecule to be revealed, for the occupation of the hot spots of the nanostructures. The protocol we used favored the coupling of the molecules with the gold nanostructures with a click reaction for both Erlotinib and the competitor molecule. With this approach the sensibility increased for low concentration of the drug because the signals of the competitor were high (see Figure 17c). The statistics intrinsic in a large number of hot spots made the protocol appropriate for quantitative results, as it was shown. Additionally, for this application naked particles like those obtained by the LAL are important because of their easy functionalization.

As a final remark, one can recall that NPs obtained by laser ablation are very useful in particular when they have to be functionalized, as it is needed for biotechnology applications, and SERS signals are used as signals for detection and diagnosis.

### 3.3. Metal Oxide Nanostructures for UV-SERS Sensing Applications

Traditionally, nanoplasmonics focuses on noble metals (Au, Ag, Cu) or their alloys whose LSPRs are in the visible (Vis) or near-infrared (NIR) spectral regions [184]. In the ultraviolet (UV) region the considerable damping due to interband transitions [185] make electromagnetic enhancements comparatively small. Nevertheless, UV nanoplasmonics may offer new opportunities in surface-enhanced Raman spectroscopy (SERS) [186], photocatalysis [187], biology [188] and public safety and security for the detection of organic molecules and hazardous organic compounds [189]. Such molecules show strong electronic absorption in the UV region, which may trigger the resonant Raman (RR) effect under UV excitation. The RR effect leads to ca. 10^8^-fold enhancement of the Raman cross-section, allowing to detect ultralow molecular concentrations [190].

Theoretical studies predict that metals with a large negative real part and a small imaginary part of the dielectric constant in the UV are suitable candidates for UV-SERS [191]. The most experimentally tested UV-SERS platforms are Al, Ga, In, Pb, Sn, Bi, Rh, Ru, Pt and Pd, owing to their availability and to the absence of interband transitions in the UV [192]. For instance, an ultrasensitive label-free detection of adenine molecules adsorbed on Al nanoparticle arrays using deep-UV SERS (with 257.2 nm excitation) was reported [193]. However, similar to Mg, Al suffers from the formation of an oxide layer several nanometers thick that limits the UV plasmonic performance. Although encapsulating the metal core within chemically inert ultrathin silica shells is a strategy to overcome this issue, it is difficult to implement given the not-easy-to-control thickness of the SiO_2_ shell [194,195,196]. Ga is interesting for its self-terminating oxide monolayer, but it has an intrinsic low electrical conductivity besides the melting point near room temperature that hinders its manipulation [197,198]. Interestingly, Bi NPs prepared by laser ablation in solution [199] display LSPRs from the near UV to the IR absorption region. By using such Bi NPs as a SERS platform, the spectra of several amino acids were obtained [199], thus indicating that Bi could be considered an interesting material for the SERS detection of biomolecules, a task that is usually pursued by means of Ag and Au nanostructures. We outline that Bi plasmons lie in the UV region, whereas the Raman experiments were carried out with visible lasers [199], namely in correspondence of the tail of the optical absorption curve. Therefore, it is not possible to fully assess whether the Raman enhancement is purely electromagnetic, or chemical.

Among the transition metals, Rh has been recently discovered as a novel, nonoxidizing plasmonic contrast agent, exhibiting UV plasmonic behavior in the region between 3 eV and 7 eV, with the advantages of its oxide-free nature [200]. Despite theoretical indications, just a few papers reported UV SERS experiments with Rh based materials; we list them below, to the best of our knowledge at the time of the present writing:

- The first SERS report is due to Bilmes et al. [201] who observed an increased pyridine Raman activity on an electrochemically roughened Rh substrate. The observed enhancement was very low because visible radiation was employed as the exciting source (far from the wavelength of the Rh SPR absorption peak);
- Lin et al. [202] reported UV-SERS on Rh nanostructured surfaces, and estimated the enhancement factor to be about 102;
- Zettsu et al. [203] published high-quality UV-SERS spectra (under 325 nm excitation) of 4-mercaptopyridine attached to sub-10 nm tripod-shaped stars of Rh;
- Watson et al. [204] reported a comparative study employing Rh tripod geometry by means of SERS, surface enhanced fluorescence (SEF) and photo-induced degradation of p-aminothiophenol (PATP) under UV and visible excitation;
- Ren et al. [205] demonstrated SERS enhancement for pyridine adsorbed on roughened Rh and Ru electrodes with 325 nm excitation;
- Li et al. [206] reported on amorphous rhodium sulfide microbowls, which were successfully designed and synthesized with the guidance of theoretical calculations and characterized by an excellent SERS performance. The amorphous structure favors efficient interfacial charge transfer, and the bowl-like shape is beneficial for photon trapping by multiple light scattering.

To date, the Rh nanostructures investigated were grown by electrochemical roughening of Rh surfaces [207] or by chemical methods using different Rh precursors [208]. The limited number of papers on UV-SERS is mainly because the application of deep-UV excitation very often leads to photo-degradation of the samples. The availability of a consistent SERS enhancement in the deep-UV spectral region would therefore allow recording Raman spectra with very low excitation power [190,209], which would be beneficial to reduce photo-degradation issues.

In the recent past, pulsed laser ablation was used to make planar homogeneous Rh thin films with thickness up to 1 µm for the realization of mirrors to be used in harsh environments [210,211]. Under proper experimental conditions, LAL allows for the production of colloidal Rh suspensions in a suitable liquid medium [212,213,214], which can be employed for the fabrication of nanostructured Rh substrates with SPR peak in the 250–320 nm region. These LAL-synthesized Rh NPs are almost spherical, and their size and density distributions are markedly affected by the liquid (water, or ethanol) selected for LAL: Rh NPs obtained in water are smaller than those obtained in ethanol. The extinction spectra of the two colloids show the contribution associated to the SPR of the Rh NPs below 400 nm but, for the samples prepared in ethanol, an additional absorption feature is observed around 340 nm, which may be ascribed to Rh-C bonds [215]. The size of the NPs is affected by the chemical interactions that occur during the ablation process, in turn determined by the liquid environment. As discussed in [212], XRD and XPS results show that ablation of Rh in water leads to the production of mixed Rh/Rh-oxide phases (RhO_2_ and Rh_2_O_3_), whereas in ethanol essentially metallic Rh NPs are produced, in agreement with [216].

**Figure 18:**
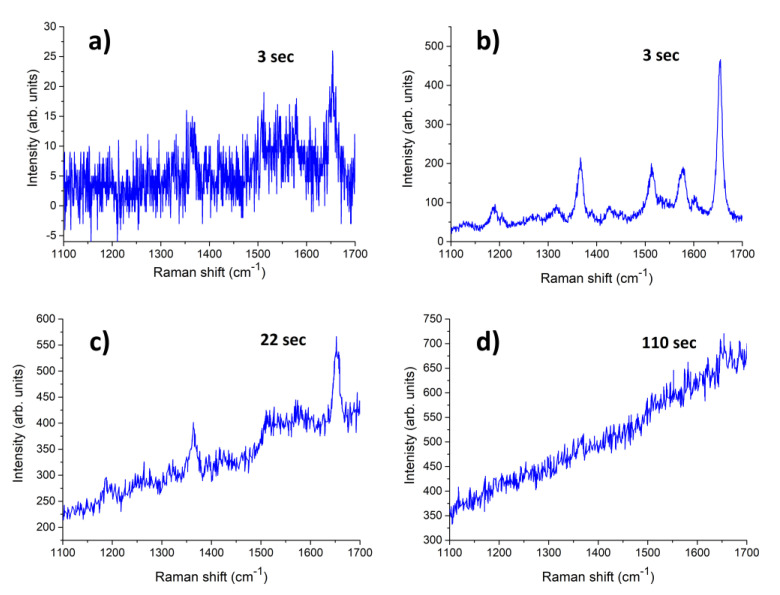
(**a**) Raman spectrum of R6G (10^−4^ M solution.) drop cast on glass and (**b**–**d**) Time-dependence of the SERS spectra of R6G (10^−4^ M solution) A drop of 10^−4^ M water solution of R6G was cast on the glass and dried in air before measurements; the Raman spectra were acquired using an HR800 micro-Raman spectrometer (Horiba, Jobin-Yvon, Longjumeau, France), using the 457.9 nm line of an Ar^+^ ion laser, setting the laser power at 1 mW. The integration times were varied from a few seconds up to 110 s to maximize the signal to noise ratio.

By fabricating a simple conductometric platform with Rh NPs produced by LAL in water, Fazio et al. [212] have demonstrated the sensing properties of Rh nanostructures toward low concentration of hydrogen in air. Here, for the purpose of showing another potential application of Rh NPs produced by LAL in water, Fazio et al. report some early results on their SERS activity by considering the reference analyte Rhodamine 6G (R6G). By comparing the SERS spectrum of R6G on Rh NPs with the Raman spectrum acquired on bare glass under the same conditions (Figure 18a,b), the resulting enhancement factor is about 20, which shows the feasibility of using Rh NPs prepared by this technique as SERS substrates. The time-dependence of the SERS signal of R6G discloses the photo-induced degradation of the analyte adsorbed on the Rh/Rh-oxide NPs under the 457 nm laser irradiation (Figure 18c,d). Remarkably, a prompt and well-resolved SERS response is observed after just 3 s of irradiation. Then, after 110 s of irradiation, the strongest characteristic peak of R6G (1650 cm^−1^) almost completely disappears (the same peak weakens by a factor of 0.4 after 22 s of irradiation). The photo-activity of nanostructured Rh surfaces was reported previously [206]; the data in Figure 18 parallel such behavior, which requires a careful control of irradiation conditions to use Rh-based NPs as a SERS platform. LAL-synthesized Rh/Rh-oxide NPs arrays suitably arranged as thin films show a moderate SERS enhancement factor. Further optimizing the deposition conditions of the NPs is likely to improve the SERS performance of such substrates in the UV spectral region, which is of relevance for biological applications.

### 3.4. Semiconductor Nanoparticles for the Degradation of Organic Pollutants

Environmental pollution and the energy crisis, with the rapid expansion of industry, human settlements and the improvement of life quality, has been, in recent years, an emergency across the entire globe. Globally, about the 80% of wastewater is discharged untreated from human agglomerations; huge amounts (millions of tons) of wastewater containing different toxic volatile (VOC) organic and inorganic pollutants (e.g., heavy metals) and toxic sludge, are released into the environment without purification treatments. Among VOC, benzene, toluene and xylenes (BTX) are the most common and dangerous contaminants in petroleum refining operations and liquid-liquid extraction processes. The copresence of these compounds in the atmosphere with NO_x_ under solar radiation generates photochemical oxidants leading to higher concentrations of ozone. Therefore, if in the past, attention was focused on water and fossil fuel resources availability, currently water-quality problems and modern green technology for energy production are mandatory. In the recent literature we find a consistent interest on group II–VI semiconductor-based NPs used to produce clean and sustainable energy sources (solar cells and hydrogen generation) and to remove organic pollutants (e.g., BTX) by photocatalysis.

Different conventional physical, chemical and biological routes, including oxidation, precipitation, solvent extraction (e.g., sulfolane for BTX), distillation, bio-remediation, filtration, extraction, photocatalysis, etc. for the degradation and remediation of both organic and inorganic pollutants are adopted. However, these traditional methods are characterized by a rather low efficiency and often high costs as well as they fail to sufficiently remove the emerging contaminants to the levels required by discharge standard or required for wastewater reuse [217,218,219]. In light of this, to improve the remediation performance, attention is being focused toward new approaches and advanced technologies such as advanced oxidation methods (AOMs) involving nanomaterials. Between them, nanophotocatalysis is one of most interesting AOM approaches. It is an environmentally friendly method, with high working efficiency in removing pollutants also under mild conditions, in reduction of byproducts, in the possibility to operate at ambient temperature and pressure with contained costs if compared with traditional degradation processes.

Since this approach is new, problems in terms of regeneration and recovery of the catalyst caused by adsorbed contaminants from nanomaterials must be evaluated. Recently, NPs or nanomaterials used for organic contaminant degradation in wastewater treatment are often implemented in filtration membranes and high surface area adsorbents also improving the reuse ability. However, despite many research efforts, the practical application of nanoadsorption and nanophotocatalysis for the removal of emerging contaminants remains limited.

Few researchers attempted wastewater treatment using nanocomposites made of carbon-based materials or metal/metal oxide NPs such as TiO_2_ [220], ZnO [221], Pd [222], Fe_3_O_4_ [223], cerium oxide and semiconductors–noble metals [224]. With the characteristics of nontoxicity, good stability and a special ability to oxidize, decompose toxic organic pollutants, resist corrosion, low cost and durability, TiO_2_ and ZnO-based NPs are the most versatile semiconductor photocatalysts [225,226]. However, they present a wide band gap (3.2–3.0 eV) that restricts their use under UV irradiation. To overcome this drawback, the self-doping of TiO_2_ with Ti^3+^ atomic defects and the coupling with noble metal NPs are the approaches proposed in order to enhance the TiO_2_ photoefficiency under visible/solar light irradiation.

ZnO has been proposed as an alternative photocatalyst to TiO_2_ as it has the same band gap energy, but exhibits higher absorption efficiency across a large fraction of the solar spectrum as compared to TiO_2_ [227]. In order to evaluate the photosensitization of ZnO and TiO_2_, Fenoll et al. [228] analyzed the photo-degradation of fungicides in leaching water using the ZnO and TiO_2_ under solar irradiation: the introduction of atomic defects such as those present in a nonstoichiometric metal oxide makes ZnO a better photocatalyst than TiO_2_ under solar irradiation.

According to theoretical results [229,230], the extension into visible range should be also obtained by introducing anionic dopants such as nitrogen [231], sulfur, fluorine species which confer a greater stability to the catalyst with respect to the conventional transition-metal dopants, besides determining a significant red shift of the band-gap into the visible range.

First reports on the photocatalytic effect of ZnO and TiO_2_ on organic and inorganic dyes under irradiation date back to the first half of the 1900s by studies conducted by Eibner [232] and Keidel [233]. The effect of TiO_2_ and ZnO on the color fading under light irradiation was deeply discussed in the years highlighting the superior photocatalytic activity sometimes of ZnO and other times of the TiO_2_ [234,235]. In particular, in 1931, Rao et al. observed the decomposition under solar irradiation of ammonia salts-based fertilizers due to the presence in the soil of TiO_2_ and ZnO [236,237,238], while the mechanism of photogenerated electron/hole (e^−^/h^+^) generation and recombination at different irradiation frequency was first discussed by Frenkel [239] and later by Goodeve [240,241].

In the second part of ‘900, after Fujishima and Honda (1972) discussed the photocatalytic water splitting on a TiO_2_ electrode [242], environmental applications (energy production and degradation of organic pollutants) using solar light and semiconductor oxides became one of the attractive topics of photocatalysis. The discovery of photoinduced water splitting together with other results such as photoinduced redox reactions of adsorbed substances on TiO_2_ by UV irradiation [243] and the decomposition of CN^−^ ions in UV-irradiated aqueous TiO_2_ suspensions [244] have significantly expanded the field of application of TiO_2_ based photocatalysts.

Since the 1980s, the overall, extensive effort has been conducted in the area of water treatment using TiO_2_, CeO_2_, ZnO, CdS, WO_3_, Fe_2_O_3_ NPs [242,245], carbon-based nanomaterials, zeolites and dendritic polymers [246] to detect and remove chemical and biological pollutants more efficiently, exploiting the high surface area and the peculiar electronic and optical properties of materials with reduced dimensionality. Ultimately, ZnO and TiO_2_ are still today the most studied photocatalysts for environmental applications owing to their chemical stability, nature friendly, availability and low cost.

It is therefore useful, first to describe the degradation mechanisms that involve them in presence of light irradiation and then to focus the directions of research related to water purification. One specific objective of the current review is to evaluate the employment of metal/nonmetal doping TiO_2_ and ZnO NPs as solar photocatalysts in water treatment. Then, a case of real (low pollutants’ concentrations) industrial wastewater is analyzed, unlike what is generally reported in the literature.

The degradation mechanism of pollutants in water on the ZnO and TiO_2_ surface is similar and is achieved by hydroxylation reactions mediated by oxygen radicals. In detail, electron (e^−^)/hole (h^+^) pairs are generated by semiconductor oxides under irradiation with specific energy; then, reduction and oxidation reactions take place on the photocatalyst surface. Reduction involving adsorbed O_2_, pollutants (P) and photo-generated electrons in the conduction band gives rise to oxidized free radicals ($O_{2}^{-\;\cdot },{\;\mathrm{HO}}_{2}^{\cdot }),$ which, in turn, degrade organic pollutants in the water. Photoholes (h^+^) in the valence band are highly oxidizing towards H_2_O and $O_{2}^{-\;\cdot }$ and produce reactive species, such as $O^{\cdot }{\;\mathrm{and}\;}^{\cdot }\mathrm{OH}$, able to degrade most of organic molecules until their complete mineralization in H_2_O and CO_2_.

Formation of intermediate species and degradation products in the photocatalysis have been deeply investigated in the earlier literature [247,248,249]. The main redox reactions are summarized by Horikoshi and Malato [250,251] as follows:

(14) $$\begin{array}{c} e^{-\;}+O_{2}\to O_{2}^{-\cdot }\\ O_{2}^{-\cdot }\left(ads\right)+H^{+}\to HO_{2}^{\cdot }\left(ads\right)\\ h^{+}+H_{2}O\to {}^{\cdot }OH\left(ads\right)+H^{+}\\ h^{+}+O_{2}^{-\cdot }\left(ads\right)\to 2O^{\cdot }\left(ads\right)\\ h^{+}+P\to P^{\cdot +} \end{array}$$

**Figure 19:**
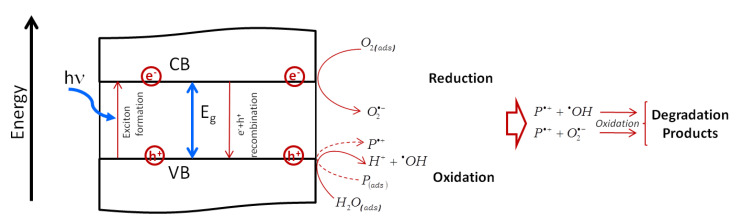
Scheme of redox reaction. CB: conduction band, VB: valence band.

Then, oxygen and pollutant radicals further react to give oxidation of organic products. A schematic of such reactions is reported in Figure 19.

ZnO and TiO_2_ unfortunately have the same drawback related to the fast electron/hole recombination that affects the photocatalytic activity. In addition, their wide energy band gap in the UV range (3.2–3.4 eV) compared to the visible light energy (1.65–2.75 eV), strongly limit a more economical application in the pollutant abatement [252,253] based on the solar irradiation (the fraction of photon energy in the UV is <5% in solar spectrum).

In an accurate and systematic study [254], the possibility of using solar-irradiated TiO_2_ for the detoxification of methyl orange (MO) as a model pollutant compound was explored. Different conditions, such as MO concentration, pH, TiO_2_ concentration and solar intensity were considered to maximize MO degradation rate. By assuming a pseudo-first order kinetics and following the relation:

(15) $$ln\frac{C}{C_{0}}=-k_{app}t$$

where *C* is the concentration at time *t* and *C_0_* is the concentration at time *t* = 0 of MO, the authors determined the apparent first order rate constants in different times with different solar intensities (Table 1). Although several studies reported the best experimental conditions to maximize the pollutant oxidation rate by using semiconductor oxides, a nanocatalyst modification was however necessary to further improve the catalytic performance under solar irradiation. As already mentioned, some of the most investigated and performing strategies to modify absorption properties of ZnO and TiO_2_ are the introduction of atomic defects and the design of new nanocomposite materials resulting by the coupling of different semiconductor oxides, metals and carbon-based materials [255,256,257,258,259,260]. The improved photocatalytic performance in the entire frequency range of the solar spectrum attained by the so-called blue Titania, namely defective TiO_2_ (bearing oxygen vacancies and under-cordinated Ti^+4^) was demonstrated [261,262] not only towards pollutants but also in the water splitting processes [257]. The enhanced performance was attributed to the charge injection/recombination dynamics [263,264].

Doping with atomic defects can be achieved by different routes, ranging from vacuum treatments, reducing thermal treatments [265,266] to hydrogen plasmas [267,268], yet one of the more convenient, flexible and environmentally friendly strategies is to use LAL of semiconductor oxide NPs dispersed in liquid (typically water). This leads to structural transformations in terms of stable under-coordinated atoms, different oxidation state of transition metal in the oxide and oxygen vacancies which play a fundamental role in the mechanism of photodegradation.

Chen et al. [269] demonstrated that the formation of Ti^3+^ species and a surface structural disorder can be induced in titania NPs by LAL: the resulting modified catalysts exhibit good photocatalytic performance under visible light towards the abatement of dyes. Moreover, Filice et al. [257] confirmed a long living and an extended catalytic efficiency in the visible spectrum for water photo-splitting by titania colloids, modified by nanosecond pulsed laser irradiation.

Another simple approach to improve the solar activity of TiO_2_ is the synthesis of TiO_2_-based composites with other transition metal oxides. In this context, photocatalytic oxidation of ethanol, as a model for VOCs, was investigated by Fiorenza et al. [270] by introducing Co oxides into TiO_2_: TiO_2_-CoO_x_ composites exhibit a maximum decrease of the TiO_2_ band gap of about 0.2 eV when 1% (w.) of CoO_x_ is added to TiO_2_ (extrapolated band gap 3.03 eV). Moreover, in the same catalyst, a further band gap at 2.3 eV was observed confirming the extended absorption in the visible range.

Recently, D’Urso et al. [259] prepared ZnO and ZnO/Au in the form of nanocolloids and nanorods by picosecond pulsed laser ablation in water. Experimental results indicated gold decorated ZnO nanorods as high photodegradation efficiency catalysts towards methylene blue (MB) dye, under UV light irradiation, as compared to bare ZnO. In this regard, doping with metals could promote photocatalysis by different concomitant events:

- A decrease in the band gap energy due to the increased structural disorder;
- A strong absorption in the visible range, due to the Surface Resonance Plasmon (SPR) of free electrons (the same effect was observed in Ag-TiO_2_ catalysts) [260,271,272], favors the injection of photo-excited SPR electrons into the conduction band (CB) of TiO_2_, thus creating separated electrons-hole pairs and hindering the recombination process [273].
- Metals can act as reservoir, promoting interfacial electron transfer processes from the semiconductor CB toward metal NPs, being the Fermi level of the metal lower than the CB of the semiconductor, leaving holes in the valence band (VB) of the photocatalyst.
- Electrons transferred from Au NPs surface to the oxide can be caught by oxygen atoms giving active O_2_^−^ species with the increase of the photo-catalytic activity [274].

In the literature, several studies reported that titania-based photocatalyst modified with Pt, Cu, Pd, Rh, Ni [260,275,276,277,278,279,280] show almost the same features in terms of efficient charge transfer phenomena, thus exhibiting a higher efficiency both in the UV and in the visible range. Ultimately, photo-activated reactions as 2-propanol degradation, chemo-selective oxidation of alcohols, efficient phenol removal from aqueous solutions by Ag in photoactivated ZnO-Ag [281] are other reported examples of enhanced efficiency in modified catalysts.

We remark that most of the studies on photo-degradation are related to dye molecules, alcohols or other molecules in the field of pharmaceuticals (ibuprofen, ciprofloxacin, etc). However, different kinds of pollutants are contained in industrial waters and wastewaters, from agricultural pollutants, inorganic pollutants (metals compounds, inorganic salts, heavy metals, mineral acids), plastic pollution, VOC. An internet search with keywords “photodegradation of volatile organic compounds” resulted in about 700 hits, compared with the 7000 for “photodegradation of MB” and 400,000 by editing the keyword “photodegradation”. Literature lacks systematic investigation/rationalization of these dangerous contaminants. Among VOC, benzene, toluene and xylenes (BTX) are the most common and unsafe compounds in petroleum refining operations and liquid-liquid extraction processes. The copresence of these in the atmosphere with NO_x_ and solar radiation generates photochemical oxidants leading to higher levels of ozone. The abatement of these contaminants is therefore mandatory.

Here we investigate the removal of BTX from contaminated industrial waters through photocatalysis by using modified TiO_2_ nanocatalysts with the introduction of atomic defects or by joining TiO_2_ with 3 wt%. AuNP. In the first case, commercial anatase TiO_2_ (Aldrich) was treated by laser irradiation in liquid, using the third harmonic (355 nm) of a Nd:YAG pulsed laser (10 ns, 10 Hz). The treatment was carried out at pH 9 for 60 min. The second modified catalysts (AuTiO_2_) was obtained, first by synthesizing Au NPs by laser ablation in water, by focusing the first harmonic (1064 nm) of a Nd:YAG on a pure gold (99%) target. Then a dispersion at 3% (w.) of Au NPs and TiO_2_ in water was prepared. All catalysts were characterized by Raman, Diffuse Reflectance and Photoluminescence spectroscopies.

**Figure 20:**
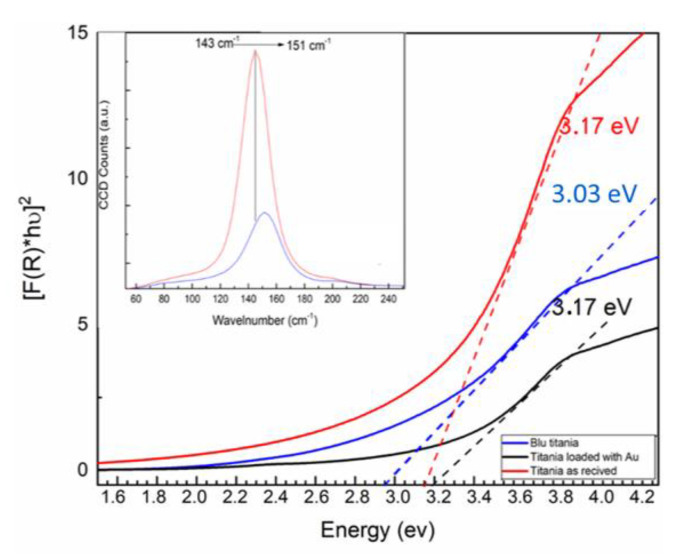
Energy bandgap calculation by Kubelka-Munka transformation for the three catalyst. Inset: a zoom of the lower energy region was set up the Au NPs adsorption (right side). In the inset is reported the micro-Raman spectra of as prepared TiO_2_ (red line) and of blue Titania (blue line) with a zoom of the TiO_2_ E_g_ anatase peaks before and after the laser treatment.

BTX aqueous solutions used for BTX photodegradation, came from an aromatic hydrocarbon extraction process that uses sulfolane as extraction solvent, in particular from a tank that collects waters from various sections of the BTX production plants, including the sulfolane extraction sections. In the Table 2 the percentage composition of employed industrial waters are reported together with the pH value. Industrial waters were then diluted until a concentration in benzene of 10^−3^ M, being the component present at higher concentration. The photodegradation of the diluted industrial waters was conducted with a solar simulator and was monitored recording the UV–Vis optical density in the 225–275 nm range [282]. Photocatalytic data showed that the modifications induced by the laser treatment of TiO_2_ led to a bandgap decrease as compared to the bare TiO_2_ due to the introduction of oxygen vacancies and defects inside theTiO_2_, as shown in Figure 20. The bandgap value, calculated by a Kubelka–Munka function (i.e., *F*(*R*) plotting (*F*(*R*)*hν*)1/*r* as a function of the photon energy (*hν*)), exhibits a strong decrease after laser irradiation, from the 3.17 eV in unmodified TiO_2_ to 3.03 eV in the laser-treated sample. It is worth to notice the inset of the same figure, where a zoom of the lower energy (2.70–1.80 eV) region is reported. In this region, the contribution of Au NPs to the optical absorption is recognizable. Au absorption feature is centered in the middle of the visible range with an energy around 1.93 eV, meanwhile there is not any adsorption in the visible range for the other two catalysts. This structure modification results in enhanced photoactivity of the laser-treated TiO_2_ sample towards the degradation of organic pollutants in industrial waters compared to un-modified TiO_2_.

To better understand the effect of laser irradiation on TiO_2_, blue titania NPs were characterized by Raman spectroscopy. In the inset of Figure 20 is reported a zoom of the E_g_ peak of the Raman spectra of as prepared TiO_2_ and of the irradiated one. Micro-Raman spectra, obtained using the 530 nm excitation wavelength, of commercial TiO_2_ agree with the typical peak position of the anatase phase (red curve). Its characteristic peak: E_g_ (144 cm^−1^) is very intense. However, after laser irradiation, a significant change of the peak intensity and a shift of anatase vibrational features is observed. Such a change is ascribed to the generation of oxygen vacancies in the atomic structure of the catalyst. The trend observed is analogous to that reported [283], in samples for which the calculated value of the O/Ti atomic ratio is reduced to 1.88 with respect to the native value of 2. We outline that the presence of oxygen vacancy defects, as already discussed, decreases the band gap value and also generates more active Ti^3+^ sites.

**Figure 21:**
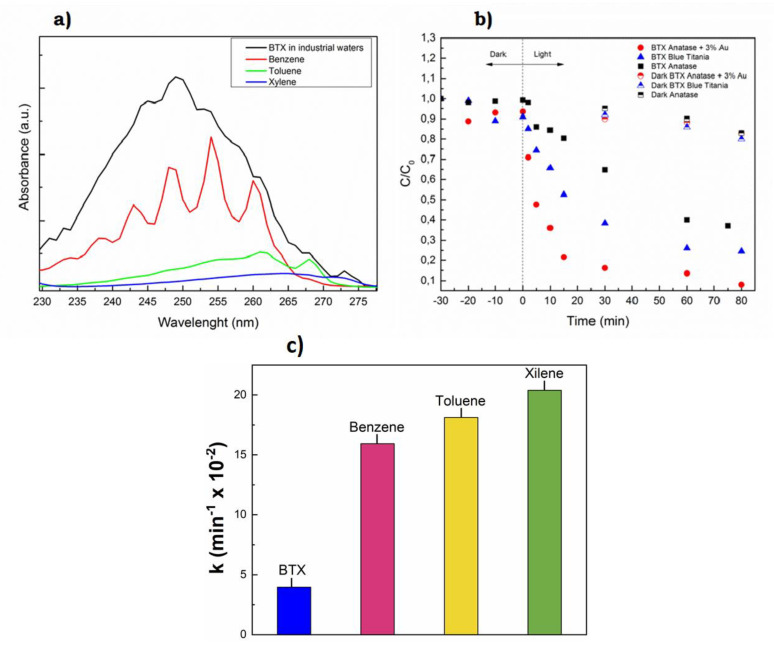
UV–vis spectra of BTX/sulfolane aqueous solution in presence of TiO_2_ laser-treated catalyst under solar lamp irradiation (**a**); calculated kinetic constant of the photodegradation for the three catalyst (**b**); kinetic constant for the photodegradation reaction of benzene, toluene and xylene in pure water (10^−3^ M) compared with the BTX mixture in presence of blue Titania catalyst, under solar light irradiation (**c**).

The UV–Vis spectrum in the 275–230 nm range of BTX aqueous solution is reported in Figure 21a together with that of pure benzene, toluene and xylene, according to the aromatic concentration reported in the table in Figure 21. By fixing the catalyst concentration in all experiments to 0.15 mg/mL, we have observed a negligible contribution of the catalyst into the BTX absorption spectra. The kinetics of disappearance of aromatic mixtures is displayed in Figure 21b (an adsorption period in the dark of 30 min). Photocatalytic degradation was performed in a closed box kept at low temperature in an ice bath to avoid evaporation of BTX from the solution, under strong stirring condition. The intensity decrease of the band at 254 nm, mainly associated to benzene electronic transitions [282], was monitored as a function of the irradiation time in a range of 80 min. In the presence of blue titania NPs, 76% of benzene, the most abundant component in the employed industrial waters, is removed after 80 min; after the same irradiation time, when AuTiO_2_ NPs were employed as photo-catalyst a residual less than 1% of benzene in water was detected. Moreover, we obtained that, after only 5 min, the percentage of BTX decreased by 53% when Au-decorated TiO_2_ is used; instead, when blue and commercial TiO_2_ are employed as a photocatalyst, BTX decreases by 26% and 14%, respectively, during the same time.

By a comparison of kinetic constants of photodegradation, evaluated by considering the peak intensity decrease at 254 nm and by using a pseudo-first order reaction kinetic model, we found an increase of *K_app_* starting from bare TiO_2_ with a value of 1.4 × 10^−2^ min^−1^ up to blue TiO_2_ of 3.8 × 10^−2^ min^−1^ and TiO_2_ loaded with Au NPs of 8.3 × 10^−2^min^−1^. It is clear that the interaction of Au with the surface of the TiO_2_ catalyst enhances the catalytic process, thus degrading more quickly almost the entire pollutant. The improvement in the efficiency of the photodegradation process is not only related to the lower value of the energy bandgap of the catalyst (blue titania presents the lowest energy BG with respect to the other two catalysts), but it seems that the main responsible of the great improvement of the photoefficiency is due to the presence of Au NPs [260,271,272], that, thanks to the SPR and high electron density, are able to enhance the electron transfer from the Au NPs to the catalyst [273,274].

To better understand the defect doping effect, blue titania was used as a catalyst to degrade single components of the BTX mixture. For this purpose, three solutions containing commercial Benzene, Toluene and Xylene, respectively, were prepared in millipore water at the same concentration of 10^−3^ M.

The photodegradation experiment was conducted under the same experimental conditions above described. The photoefficiency of blue titania towards the degradation of single aromatic compounds (benzene, toluene and xylene) 10^−3^ M in millipore water are compared in Figure 21c. After 20 min, all pollutants reduced by 95% with respect to the initial concentration. It is to be noted that the lowest kinetic constant is obtained for the degradation of benzene. However, this value results three times higher than that obtained for BTX in industrial waters. It seems [284] that benzene degrades less quickly when it is mixed with toluene. This finding could explain the difference in the kinetic rate and the lower value of kinetic constant related to the degradation of the BTX mixture, in which benzene is the most abundant component and toluene the second one.

## 4. Conclusions

In this review, we reported on various laser-based methods for the generation of colloids with a tight control on their properties (the purity of the product, the selectivity in the crystal structure of the oxide, a fine control of the particle size distribution). The peculiar chemical–physical properties that new nanomaterials produced by LAL can offer have been described in view of specific technological applications and showed to be competitive with those of conventional chemical procedures. However, LAL colloids are not yet applicable on a large scale. To reach this purpose, we considered it essential to analyze in depth the fundamental LAL mechanisms, taking into account the multiple time-scale transient processes that take place under the different experimental conditions which, ultimately, determine the nanocolloids properties.

## 5. Outlooks

Since about 2010 there has been a growing research and development of third-generation nanotechnologies, which are characterized by their multiscale architecture (i.e., involving macro-, meso-, micro- and nanoscales together) and three-dimensionality. Some applications driving the developments of third generation nanotechnologies are biosensors or drug-delivery vectors modelled on biological templates. After 2015, the fourth-generation nanotechnologies employ “molecular manufacturing”, to achieve multifunctionality and the control of functions at a molecular level. The global deterioration of water, soil, and the atmosphere by the release of toxic chemicals from the ongoing anthropogenic activities is becoming a serious problem throughout the world. Thanks to the remarkable advances in nanotechnology and the urgent need to develop green, robust and economic approaches for innovative biomedical purposes and environmental remediation, LAL-prepared nanomaterials could be successfully used in biomedicine, in wastewater treatment systems, for the production of clean and sustainable energy sources (e.g., solar cells and hydrogen generation systems) and for the removal of organic pollutants by photocatalysis.

Despite their great potential, some aspects of the LAL processes and of their products need to be optimized. Strong efforts should be devoted to achieve higher colloidal stability, preventing aggregation phenomena and the formation of larger particles, which, for instance, affect the catalytic properties and could even lead to the inactivation of the material. Efforts should also be devoted to the embedding of plasmonic NPs in bulk-heterojunction solar cells to promote photo-conversion. Furthermore, to achieve the gram-scale production that is required for industrial applications it is essential to properly define a LAL procedure and protocol.

Although LAL-synthesized NPs are referred to as ligand/surfactant-free, they are not totally naked and still bear their own surface chemistry, i.e., some of the NP surface atoms are oxidized or complexed. Presently, an appropriate protocol to realize performance-oriented colloidal synthesis and processing in order to prepare more isochronous surface modification in metal and semiconductor NPs (also within a polymeric matrix) is still not defined. The latter is an urgent request of modern manufacturing based on laser ablation to make possible in-situ engineering, thereby opening the way to fabricate functionalized (or coated) NPs.

Moreover, nanostructures have dimensions which are similar to those of subcell structures, like receptors, and for this reason they can strongly interact. This is an important opportunity which can be exploited, as above reported, using nanostructures with engineered functionalizations, for a theranostic targeted nanomedicine. Indeed, since NPs produced by LAL have appealing and controlled plasmonic properties, spanning from the visible to the UV region, they are excellent platforms for SERS applications, even though significant efforts should be spent to obtain a higher signal-to-noise ratio in the UV spectral region. To this purpose, further studies closely combining the features of LAL with the application demands of various functional materials are still necessary.

## Figures and Tables

**Table 1:** Results on detoxification of methyl orange (MO) as under different conditions (MO concentration, pH, TiO_2_ concentration and solar intensity). Reprinted from Ref. [254], Copyright (2002), with permission from Elsevier.

Rate Constant k of Degradation of TiO_2_ (h^−1^) in Different Time with Different Solar Intensities W h/m^2^			Effect of Concentration on the Degradation Rate (h^−1^) during April 2001				
Month	1st Rate Constant	Solar Intensity	[MO] April 2001	*k* (465 nm)	*R* ^2^	pH before Reaction	pH after Reaction
July 1999	0.6195	4480	2 × 10^−3^ M	0.0412	0.94	6.4	6.1
November 1999	0.367	2535	4 × 10^−4^ M	0.1329	0.96	6.4	6.0
December 1999 (cloudy)	0.2455	2177	8 × 10^−5^ M	0.2010	0.98	6.5	6.1
January 2000	0.3314	3060	4 × 10^−5^ M	0.6393	0.92	6.5	6.4
February 2000	0.6489	3419	1 × 10^−5^ M	0.2912	0.90	5.9	6.2
March 2000	0.7953	4255					
May 2000	0.5445	4814					
June 2000	0.4645	3969					
October 2000	0.3491	3970					

**Table 2:** Percentage composition and pH of employed industrial waters.

pH	9.1
Benzene	90.3%
Toluene	6.1%
m-xylene	0.85%
o-xylene	0.46%
p-xylene	0.32%
Etylenbenzene	1.32%
Aromatics	1060 mg/L
